# Growth rates of coral reefs peaked at 25 °C through the Holocene

**DOI:** 10.1371/journal.pone.0342527

**Published:** 2026-03-11

**Authors:** Tonya Macedo, Robert van Woesik

**Affiliations:** Institute for Global Ecology, Florida Institute of Technology, Melbourne, Florida, United States of America; Living Oceans Foundation, TAIWAN

## Abstract

For millennia, corals have built coral-reef structures upon the remains of past generations of coral skeletons, forming the world’s most diverse marine ecosystems. Yet, ocean warming and regional and local disturbances are reducing the capacity of coral reefs to grow and keep pace with sea-level rise. Understanding which environmental and climatic conditions influenced reef growth in the past, when human populations were small, may help us understand how reefs respond to contemporary environmental changes. Using coral cores dating back 11,700 calibrated years before present (yr BP) from 291 sites across the Pacific, Indian, and Atlantic Oceans, we examined the relationships between seven environmental and climatic variables and coral-reef growth using a spatial-temporal Bayesian mixed model and a deep-learning neural-network analysis. Our results show a positive relationship between the rate of change in sea level and reef growth. Reef growth responded nonlinearly to sea-surface temperature, peaking at ~25 °C, during the Holocene Thermal Maximum, between ~7,000 and ~5,500 yrs BP. During this period, atmospheric carbon dioxide (CO_2_) concentrations were ~325 parts per million (ppm) by volume. Our findings reveal that atmospheric CO_2_ levels currently exceeding 335 ppm, combined with sea-surface temperatures and modern marine heatwaves, are less than optimal for contemporary coral-reef growth, inhibiting their ability to keep pace with sea-level rise.

## Introduction

For the past 400 million years, when environmental conditions were favorable, corals built extensive carbonate structures, supporting Earth’s most diverse marine assemblages. The growth rates of coral reefs have long been known to be a function of ocean temperature, rate of sea-level rise, and available irradiance [1–5]. Even today, coral reefs rarely grow in localities where temperatures decline below 18 °C for more than three months a year [2]. At the other extreme, corals have locally adapted to high sea-surface temperatures, such as in the Persian (Arabian) Gulf, where modern summer temperatures are typically between 30 and 34 °C [6]. However, recent marine heatwaves have caused unprecedented temperature anomalies, resulting in extensive coral mortality worldwide, including the Persian (Arabian) Gulf, where temperatures have frequently exceeded long-term averages [6]. These observations suggest that modern coral reefs may already be approaching environmental limits beyond which reef growth will cease [7–9]. Here, we examine the relationship between rates of coral-reef growth and major oceanic and climate fluctuations spanning the past 11,700 years before present (yr BP; 1950 Common Era [CE]) through the Holocene. This study will help gauge the response of modern coral reefs to contemporary and future environmental change.

**Fig 1:**
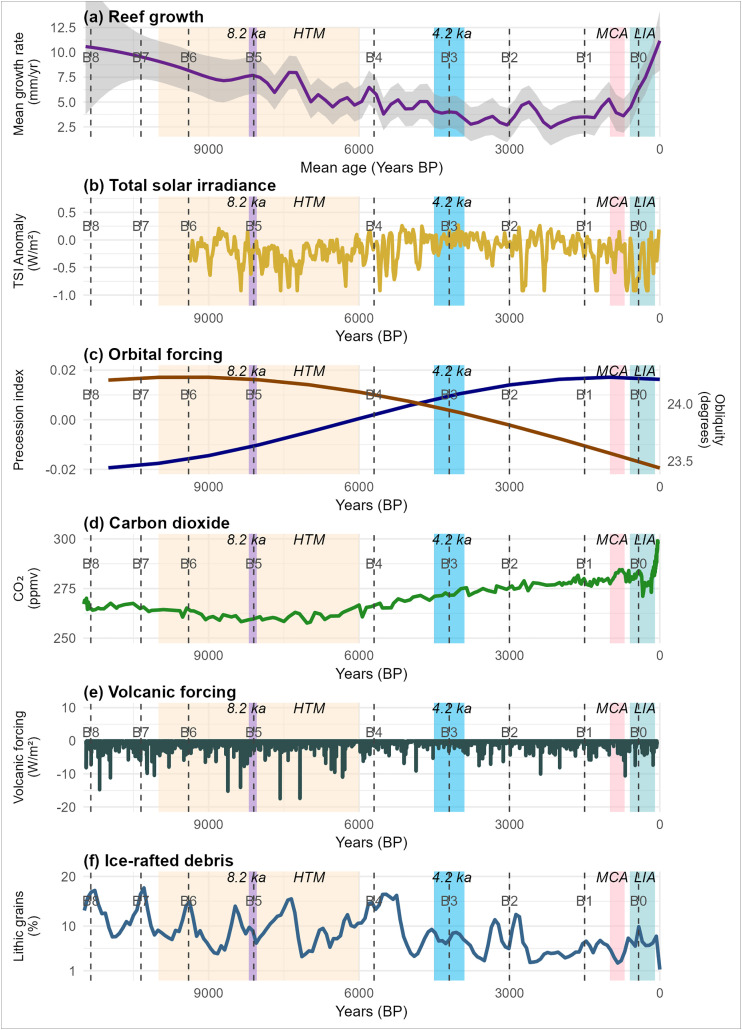
Five global environmental and climatic variables and mean coral-reef growth over the past 11,700 calibrated years before present (yr BP; 1950 Common Era [CE]), as reported through five major temperature-fluctuation events of the Holocene. The ecological response of mean reef growth rates (mm/yr) **(a) reef growth** (mm/yr) is represented by a dark purple line [24]. The five global variables include: 1) radiative forcing of **(b) total solar irradiance** (TSI) represented by the yellow line [25], where the TSI anomaly is relative to 1365 W/m^2^; 2) two orbital parameters of **(c) orbital forcing** represented by the orange line of Earth’s axial obliquity (degrees) and the blue line of orbital precession, e × sin(ϖ), where *e* is the eccentricity, and ϖ is the longitude of perihelion, which is dimensionless because it is a product of two ratios; 3) the first atmospheric-composition variable of **(d) carbon dioxide** (CO_2_) in parts per million by volume (ppmv) represented by the green line [26]; 4) the second atmospheric-composition variable of **(e) volcanic forcing** (W/m^2^) calculated from the mean stratospheric aerosol optical depth represented by the black line [27]; and 5) **(f) ice-rafted debris** (% of lithic grains) represented by the blue line [28]. The five major temperature-fluctuation events of the Holocene depicted are: 1) the Holocene Thermal Maximum (**HTM**) (10,000–6,000 yr BP); [13], shaded in yellow, 2) the **8.2 ka** yr BP cooling event (8,200–8,040 yr BP) [14], shaded in purple, 3) the **4.2 ka** yr BP cooling event [4,500–3,900 yr BP [15]], shaded in blue, 4) the warm Medieval Climate Anomaly (**MCA**) (1,000–700 yr BP) [16], shaded in pink, and 5) the Little Ice Age (**LIA**) (600–100 yr BP) [16], shaded in light green. The nine **Bond-cycle events** [29] are dashed gray lines labeled from B0 to B8, where B0 is the most recent cycle.

Past climate fluctuations have induced changes to marine ecosystems [10], and coral reefs are no exception [1,3]. For example, in the North Atlantic, from the late Pleistocene to the Holocene, ice-laden water intermittently drifted south from Greenland, cooling the ocean, reducing salinity, and blocking ocean circulation [11]. This change in ocean circulation reduced northward heat transport, causing global cascading effects on oceanic and atmospheric conditions. Many biological records from trees, planktonic foraminifera, corals, and fossil pollen align with changes in North Atlantic glacial ice [12]. Paleo-records suggest that over the past 11,700 yr BP, the Holocene was characterized by at least five significant temperature fluctuations: (i) the Holocene Thermal Maximum (10,000–6,000 yr BP) [13], (ii) the 8.2 ka yrs BP cooling event (8,200–8,040 yr BP) [14], (iii) the 4.2 ka yrs BP cooling event (4,500–3,900 yr BP) [15], (iv) the warm Medieval Climate Anomaly (1,000–700 yr BP) [16], and (v) the Little Ice Age (600–100 yr BP) [16] (Fig 1). The Holocene Thermal Maximum was a warm phase with high rates of reef growth [3,17–19], and is considered a consequence of Earth’s orbital patterns involving eccentricity, tilt, and precession (Fig 1). This period led to increased solar radiation and warmer temperatures, which varied regionally (S1 Fig in S1 File) during the middle part of the Holocene, and coral-reef growth was particularly high [3,20,21]. By contrast, the 4.2-ka cooling event shut down reef growth off the coast of Panama for 2,600 years [22], although the reefs in the South China Sea showed only minor changes over the same period [23].

Similarly, coral reefs are sensitive to sea-level changes [3,5,30] because small increases in sea level can provide accommodation space on reef flats [31], increasing coral growth. However, large, rapid changes in sea level can reduce irradiance, suppressing coral growth. At the end of the last ice age, global sea level was 130 m lower than today. Deglaciation caused sea level to rise rapidly until ~5,500 yr BP, when sea level stabilized in the Indian and Pacific Oceans, although there was considerable geographical variation in regional response rates (S2 Fig in S1 File) [32]. By contrast, sea level did not stabilize in the Atlantic Ocean until recently, ~ 50 yr BP, because of continental rebound effects from the heavy ice on the North American continent during the last ice age [32] (S2 Fig in S1 File). During the deglaciation period, coral reefs grew either rapidly to keep up with the rising sea levels, progressively caught up after sea level had stabilized [3,33], or drowned [5]. Since sea-level stabilization, coral reefs in the Pacific and Indian Oceans have propagated laterally and formed large reef flats between the latitudes ~29 degrees north and south of the Equator [34]. Beyond 29 degrees, modern coral reefs appear as isolated coral assemblages without developed reef flats; for example, in Japan [35] and eastern Australia [36,37].

Examining the recent history of coral-reef growth, past environmental conditions, and climate cycles may help explain the present response of coral reefs to current trends in ocean warming and other disturbances. A global analysis of geological coral-reef cores through the Holocene may also provide insight into how environmental and climatic variables previously influenced coral reefs. The objective of our study was to determine which environmental and climatic variables influenced coral-reef growth rates through the Holocene and which ocean temperatures were optimal for reef growth. This study highlights how coral reefs responded to past climatic changes and provides insight into how they are expected to respond in the near future.

## Methods

### Coral-reef growth

Coral skeletons are well preserved in the fossil record because of their hard calcium carbonate (CaCO_3_) composition. To infer past reef conditions, geological assessments of coral reefs extract geological cores and apply radiocarbon dating and reservoir corrections to determine the age of reef initiation and growth rates (i.e., growth rates in mm/yr). The RADReef database [24] consolidated 3,535 radiocarbon-dated geological core points to calculate 1,954 independent mean growth rates (mm/yr), using 950 geologic cores at 347 unique coordinates, hereinafter referred to as sites.

**Fig 2:**
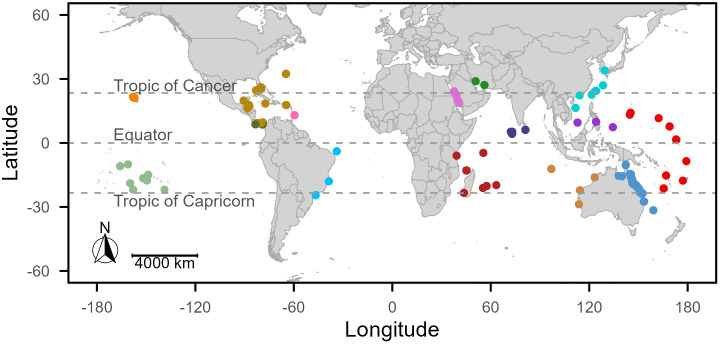
The location of 1,890 samples, dating back 11,700 calibrated years before present (yr BP; 1950 Common Era [CE]) through the Holocene from 291 sites across the Pacific, Indian, and Atlantic Oceans, colored by region. The 15 regions (from left to right) include: 1) Hawaii in orange, 2) French Polynesia in light green, 3) the Caribbean in light brown, 4) Barbados in pink, 5) the eastern Pacific in dark gray, 6) South America in light blue, 7) the Red Sea in light purple, 8) the Persian (Arabian) Gulf in dark green, 9) the southwestern Indian Ocean in maroon, 10) the Indian Ocean in navy blue, 11) Asia in teal, 12) the Sunda Shelf in dark purple, 13) northwestern Australia in gold, 14) the central Pacific Ocean in red, and 15) eastern Australia in medium blue. The map was generated using world polygon data from the *maps* package [38] in R [39].

For this study, we used these mean growth rates, as calculated and reported in RADReef [24], as the response variable. The reef growth rates (mm/yr) were calculated within a given core as the length of the core section between two different time stamps, T_1_ and T_2_, defining the sample interval over which reef growth was calculated. In cores, where more than two dates existed, the T_2_ from one interval also serves as the T_1_ for the subsequent interval. We reconstructed these T_1_–T_2_ pairs within cores to calculate the core-section interval in years, which allowed us to assess the amount of growth over the temporal resolution of each sample. We excluded any samples with T_2_ ages greater than 11,700 years, retaining 1,950 samples. Reef growth rates ranged from <0.1 to ~560 mm/yr and were right-skewed, with upper values exceeding biologically realistic growth limits for coral reefs. To address this issue, a two-tailed 97th percentile filter was applied to remove extreme outliers (S3 Fig in S1 File). This removal resulted in a final dataset of 1,890 samples ranging from ~0.11 to 54.7 mm/yr, from 691 cores at 291 sites (Fig 2). Of the 691 geologic cores, 436 contained more than one sample within the core, and the highest number of samples within a single core was 26 samples. To account for repeated core names across studies and to link samples to their respective cores, a unique core identifier (Core ID) was created by combining the study name with the reported core name.

The final dataset covers a temporal range from 11,635 years before present to 57.9 years after 1950 (~ 2008 Common Era [CE]). The core segment lengths, which are the number of years between T_1_ and T_2_, had a median core segment length of ~410 years. We assessed potential shifts in modern site locations by calculating the mean age and the mean difference between the T_1_ and T_2_ ages for each sample using the *palaeoverse* package [40] in R. Across five spatial reconstruction models, the maximum geodesic displacement in modern locations since the mean age was ~ 1.2 km—small relative to the resolution of the environmental variables—so modern latitudes and longitudes were used for data extraction (S4 Fig in S1 File). Notably, Barbados was treated as a separate region because its coral reefs develop on a tectonically active and unique setting [32]. Therefore, Barbados was excluded from the observational assessment of regional reef growth trends through time.

### Solar insolation

Corals support internal symbionts that photosynthesize, supplying the coral with energy-rich resources that support coral growth [41]. The rate of symbiont photosynthesis is governed by irradiance, specifically the 400−700 nm wavelength referred to as photosynthetically available radiation (PAR). Although a historical PAR-specific dataset with sufficient temporal coverage is not available, Lean (2000) demonstrated that total solar irradiance (TSI) (~300 nm to 3,000 nm) — the incoming solar radiation received at the top of Earth’s atmosphere [42] — is almost identical to narrower wavelengths between 400–1000 nm. Therefore, TSI was used as a proxy for photosynthetically available radiation. We utilized TSI data (W/m^2^) from Steinhilber et al. [25] and the *palinsol* package [43] in R to examine the spatial and temporal influence of solar insolation (W/m^2^) — the incoming solar radiation received at Earth’s surface [42] — on mean coral-reef growth rates. Using a physics-based model, Steinhilber et al. [44] reconstructed a 9,389-year proxy of total solar irradiance from Beryllium-10 (^10^Be) ice-core records extracted from Greenland and Antarctica. TSI values were originally reported as anomalies and were converted to absolute values relative to the reference value of 1,365 W/m^2^. Because the reconstruction years did not align with each sample’s mean age, TSI was estimated at the T_1_ and T_2_ sample ages via linear interpolation, weighted by temporal distance, using the *approx* function in R. While interpolation may reduce potential short-term variability, it is unlikely to alter the overall response. TSI was interpolated at 22-year increments, the mean temporal resolution of the TSI record, across the ages of the core segments. For example, if a core segment, between T_1_ and T_2_, was 200 years, there would be nine age points where TSI was interpolated. If the core segment was less than 22 years, we used TSI at the endpoint ages only. These TSI values represent solar irradiance at the top of the atmosphere, which we then converted to site-specific, surface-level solar insolation (W/m^2^), using the *Insol* function in the *palinsol* package [43] in R.

The *Insol* function computes mean daily surface-level insolation as a function of TSI (top-of-the-atmosphere), latitude, solar longitude, and sun-hour angle. In this computation, latitude accounts for the solar zenith angle, which controls the flux of radiation at the surface. Additionally, the solar longitude, relative to the vernal equinox (day 80), accounts for seasonal variation in photoperiod. The *Insol* function used the orbital parameters from the La2004 solution [45] to calculate solar insolation (W/m^2^) at the site’s latitude, applying the Berger [46] formulation at 22-year increments across the growth period.

Monthly solar insolation (W/m^2^) was estimated at the midpoint of each month and averaged to an annual value using month-length weights to account for differences in the number of days per month. The mean annual solar insolation (W/m^2^) for each sample was calculated by averaging the annual values across all age points within each sample. These mean annual solar insolation values were used as the representative irradiance for each sample. Thirty-three samples (~1.7% of the total) had ages completely outside the TSI record (9,389–0 yr BP) and were extrapolated using the nearest available measurement using the *approx* function in R [39]. Over 97% of samples had ages entirely within the TSI record, while samples partially overlapping the record were calculated using only the age points within the available TSI range. This approach maintains the full sample size while utilizing the highest possible temporal resolution.

### Orbital forcing

Orbital forcing parameters, obliquity, precession, and eccentricity, cause variance in the distribution of solar insolation (W/m^2^) across latitudes [45,47]. Earth’s obliquity, which is the tilt of the Earth’s axis, varies across 41,000-year cycles, from 22.1 to 24.5 degrees, and is currently at ~23.5 degrees [48]. Higher obliquity amplifies seasonality at latitudes farther from the Equator, resulting in more extreme summers and winters [49]. Consequently, obliquity affects solar insolation (W/m^2^) at high latitudes more than at low latitudes. By contrast, precession—the wobble of Earth’s axis—primarily affects the mid-latitudes and equatorial regions, where precession cycles influence atmospheric circulation, rainfall patterns, and ocean-surface conditions [50]. Because eccentricity, the shape of Earth’s orbit, modulates the amplitude of the precession signal, paleoclimate studies often use the precession climate index, calculated as *eccentricity × sin*(ω), where *ω* is the longitude of perihelion in radians [45,46]. Orbital solutions for obliquity, precession, and eccentricity were retrieved using the *astrochron* package [51] in R, and the precession index was calculated to represent climatic precession from the provided eccentricity and precession values. Because orbital forcing occurs on long temporal scales, the precession index and obliquity values were interpolated, weighted by temporal distance, using the *approx* function, based on the mean age. The ranges for precession index and obliquity estimates were ~0.04 and ~0.79 degrees, respectively.

### Atmospheric composition of CO_2_

Atmospheric carbon dioxide (CO_2_) concentrations affect oceans through both warming and acidification. High concentrations of atmospheric CO_2_ absorb and retain long-wave radiation, preventing heat from escaping Earth’s atmosphere and oceans. Chemically, atmospheric CO_2_ dissolves into ocean surface waters, forming carbonic acid that dissociates, lowering pH and reducing carbonate ion concentrations [52]. This study utilized the CO_2_ reconstruction from Bereiter et al. [26] to investigate the impact of atmospheric CO_2_ on reef growth rates throughout the Holocene. Bereiter et al. [26] utilized recent Antarctic ice-core records from the European Project for Ice Coring in Antarctica (EPICA) Dome C. The temporal resolution of the reconstructed CO_2_ data was right-skewed, with a median of ~8 years. We used the *approx* function in R, weighted by temporal distance, to estimate CO_2_ values at 8-year increments. The sample-level mean CO_2_ was the mean of all interpolated values. Because these CO_2_ estimates are global and not spatially fixed, CO_2_ was included as a random walk in the model.

### Volcanic forcing

Volcanic eruptions inject large amounts of sulfurous gases into the atmosphere, causing atmospheric and oceanic cooling that can last a decade or more. During these events, sulfur falls to the Earth and is preserved in ice, which has been converted to estimates of stratospheric aerosol optical depth — a measure of light attenuation induced by aerosol particles in the stratosphere [53]. To assess the relationship between volcanic forcing and mean coral-reef growth rates, we utilized stratospheric aerosol optical depth (SAOD; unitless; referenced at 550 nm wavelength) data from the HolVol 1.0 dataset [27]. SAOD quantifies the reduction in incoming solar radiation from scattering and absorption by stratospheric sulfate particles [53]. HolVol 1.0 provides modeled SAOD with sub-decadal dating precision, derived from sub-annual measurements of sulfate concentrations in ice layers from four Greenland and Antarctic ice cores, sampled at monthly intervals for the last 11,500 years [54]. The bipolar ice-core network enables the spatial resolution of eruptions by hemisphere and latitude, based on the estimated magnitude and location of each event.

Multiple eruptions in close succession can have cumulative effects that produce long-term climate anomalies, such as the 8.2 ka yr BP cooling event and the Little Ice Age [55]. To account for multiple eruptions, we calculated a rolling 20-year cumulative mean of volcanic forcing for each sample. In this approach, the annual volcanic forcing values within the core segment of each sample were summed over consecutive 20-year increments, resulting in a series of cumulative measurements across the core segment’s age. To calculate this metric, the HolVol 1.0 monthly SAOD values were aggregated to an annual resolution and converted to volcanic forcing using a scaling factor of 25 W/m^2^ per unit SAOD, consistent with previous studies [54,56]. For each sample, annual volcanic forcing values were linearly interpolated to the sample’s latitude using the *approx* function to account for the 10-degree spatial resolution relative to local site variability in our dataset. The annual volcanic forcing values were summed over 20-year overlapping windows throughout the core segment ages. The mean of the 20-year cumulative volcanic forcing windows was used as the sample-level metric. This rolling-mean approach quantified sustained volcanic forcing by capturing multi-year peaks, reduced sensitivity to single-year variability, and avoided boundary effects from non-overlapping bins.

### Sea-surface temperature

To assess the relationship between sea-surface temperature (SST) and coral-reef growth, reconstructed SSTs (^o^C) were obtained from Osman et al. [57], gridded as the Last Glacial Maximum Reanalysis (LGMR) dataset available via the National Oceanic and Atmospheric Administration (NOAA) Paleoclimatology Data Archive. Osman et al. [58] assimilated marine and terrestrial paleoclimate records to generate spatially and temporally continuous SST estimates over the past 23,000 years. While differences in SST estimates have been noted between proxy-only datasets and modelled reconstructions [59], Osman et al. [57] address these concerns by utilizing a wide range of proxies, data assimilation, and physics-based models. This approach reduces seasonal and depth-related biases induced by individual proxy records. The Osman et al. [57] dataset provides global coverage at ~1° × 1° spatial resolution and 200-year temporal resolution, allowing for site-specific extraction of past SST conditions. We extracted SST values for each sample, identifying the nearest valid SST grid cell using a k-nearest neighbor approach, as the number of sites did not always align within a cell containing a valid SST value. The SST NetCDF file was sparse near the Persian (Arabian) Gulf and northern Australia (S5 Fig in S1 File). SSTs were interpolated using the *approx* function in R at each T_1_ and T_2_ age. The sample-level mean SST was calculated by averaging SSTs throughout the core-segment length. Regional mean sea-surface temperatures through the Holocene are presented in S1 Fig in S1 File.

### Rate of change in sea level

Corals are sensitive to sea-level changes (mm/yr) because the depth of the reef relative to the ocean surface affects the amount of solar radiation reaching the reefs. Sea level varies regionally over time because of glacial events, glacial isostatic adjustment, tectonic activity, regional oceanographic processes [32], and variations in river discharge volume [60]. Rates of change in sea level were calculated by digitizing and compiling relative sea levels from regional sea-level datasets [61–74]. Regional sea-level curves are presented in S2 Fig in S1 File. Relative sea level was inverted to maintain consistent directional logic with reef-depth data so that positive values correspond to deeper reef submergence. This ensures that a positive rate of change in sea level represents a rising sea level and that a negative rate of change in sea level represents a falling sea level. To calculate the rate of change in sea level, annual values were interpolated for all years between the T_1_ and T_2_ ages using linear interpolation with the *approx* function in R. The mean of the resulting year-to-year differences was used as the sample-level rate of change in sea level.

### Ice-rafted debris

Bond cycles are millennial-scale drift-ice indices in the North Atlantic Ocean, identified by Bond and colleagues using composite records of ice-rafted debris derived from multiple sediment cores [29]. These indices quantify the relative abundance of three ice-rafted debris components—hematite-stained grains, detrital carbonate, and Icelandic volcanic glass—expressed as total ice-rafted debris (% of total lithic grains). Bond cycles capture centennial- to millennial-scale variability in iceberg flux associated with ice sheet instability. Peaks in the ice-rafted debris indices correspond to cooling episodes associated with broader climatic phenomena, including reduced solar irradiance, glacial advances, and suppressed tree growth in mid-latitude records. The increased ice-rafted debris during these cycles represents enhanced glacial transport, which causes changes in tropical heat export, alters atmospheric circulation patterns, and shifts the position of the Intertropical Convergence Zone [29]. Shifts in the Intertropical Convergence Zone have had far-reaching regional climatic impacts, including changes in the timing and intensity of monsoon rainfall in Asia, Africa, and the Americas [75].

To assess the relationships between Bond cycles and reef growth, we obtained ice-rafted debris indices from the North Atlantic Holocene dataset published by Bond et al. [28], compiled by Evans and Muscheler as centennial-resolution values. The mean temporal resolution of their dataset was 70 years. We used linear interpolation to estimate ice-rafted debris indices at 70-year intervals within each core sample. The sample-level mean ice-rafted debris index was calculated by averaging all interpolated ice-rafted debris index estimates during the core-segment length. Because the ice-rafted debris indices reflect a North Atlantic signal, they do not vary spatially, and therefore, the percentages of lithic grains were included as a random walk in the model.

### Data analysis

We assessed the potential correlation among the predictive variables before data analysis using the *corrplot* package in R [76]. Absolute latitude (⁰N or ⁰S), total solar insolation (W/m^2^), and mean sea-surface temperature (⁰C) were correlated greater than 0.7 (|r| > 0.7; S6 Fig in S1 File). To account for these correlations, three models were run comparing the effects of these variables on the response. Consequently, sea-surface temperature was retained as a fixed effect in the final model, whereas absolute latitude (⁰N or ⁰S) and solar insolation (W/m^2^) were excluded to avoid multicollinearity and inflated-parameter uncertainty. Two additional environmental variables were included as fixed effects: (i) cumulative volcanic forcing (W/m^2^) calculated from the mean stratospheric aerosol optical depth and (ii) the rate of change in sea level (mm/yr). Mean coral-reef growth rates data (mm/yr) were continuous, always positive, and right-skewed; therefore, we used a gamma distribution to characterize this response variable in our model. To analyze the data, we used a general linear mixed model with Integrated Nested Laplace Approximation [INLA; version 24.12.11, [77]].

To account for spatial effects, the study domain was modeled as a continuous Gaussian random field, assumed to follow a Matérn covariance structure that relates spatial correlation to the distance between points across the study domain [78]. To make inference computationally feasible, the continuous Gaussian random field was approximated by a Gaussian Markov Random Field using the Stochastic Partial Differential Equation approach. The Stochastic Partial Differential Equation framework solves for a continuous Gaussian random field by representing the field as a finite-element approximation defined by a triangular mesh, which discretizes the process across the study area [78]. The triangulated mesh was constructed with finer triangles in regions of high site density and coarser triangles in regions of low site density (S7 Fig in S1 File). This mesh-based approach, combined with the Stochastic Partial Differential Equation framework [79], provides the mathematical link between the discrete Gaussian Markov Random Field and the approximated continuous Gaussian random field. The resulting continuous Gaussian random field captures all spatial processes that can affect variation in mean coral-reef growth rates (mm/yr), effectively identifying the strength of the effects and the residual spatial variation, or latent effects, which are not explained by the observed data. In addition, we included ocean basin, oceanic region, and locality as independent and identically distributed (iid) random effects. This approach assigns an unstructured random effect to each level of the grouping factor, enabling the model to estimate variance for separate random effects. The use of independent and identically distributed random effects provides a unified modeling approach, borrowing strength from data across all locations and retaining statistical power.

Two environmental variables, carbon dioxide (CO_2_) and ice-rafted debris, lacked spatial variance and, therefore, without inflating degrees of freedom, these variables, along with two temporal covariates, mean age and core segment length, were modeled as second-order random walks in the model. These variables were discretized into bins to allow the random walks to estimate smooth, nonlinear effects across their ranges. The number of bins for each variable was selected to balance resolution and statistical robustness [80,81] (S1 Table in S1 File). The unique core identification, the study name, and the core name were included as an independent and identically distributed (iid) random effect to account for repeated samples within a single core. Obliquity and precession index were excluded from the analysis because of correlations with the mean age of 0.99 and 0.97, respectively.

### Model selection and validation

Model comparison was performed using the Watanabe-Akaike Information Criterion (WAIC) to evaluate model fit and complexity. Sea-surface temperature (⁰C) consistently exhibited the lowest WAIC among candidate predictor variables, indicating the best balance of fit and parsimony (S2 Table in S1 File). The three variables considered as fixed effects were: (i) rate of change in sea level (mm/yr), (ii) sea-surface temperature (SST) (⁰ C), and (iii) cumulative volcanic forcing (W/m^2^) calculated from the mean stratospheric aerosol optical depth. To validate the models, we performed leave-one-out cross-validation and utilized probability integral transform statistics (S8 Fig in S1 File).

In addition, a deep-learning neural-network model was developed using the H2O framework in R [82] to predict nonlinear relationships between mean coral-reef growth rate (mm/yr) and the key environmental variables: SST (⁰C), rate of change in sea level (mm/yr), and absolute latitude (⁰N or ⁰S). The dataset for the deep-learning model was partitioned into a 70/15/15 split for training, testing, and validation, respectively. Six deep learning model configurations were evaluated, varying in the number of hidden layers, the number of neurons per layer, and the number of training epochs (S3 Table in S1 File). Thirty iterations of each configuration were fit, and the final model configuration and run number were selected based on the root mean-squared error results on the validation dataset (S4 Table in S1 File). The final deep-learning neural-network structure consisted of two hidden layers, with the first containing 20 neurons and the second containing 10 neurons each. It was trained for 100 epochs with 5 rounds of early stopping. All the data and R scripts are available at: https://github.com/InstituteForGlobalEcology/Holocene_reef_growth and on Figshare (https://doi.org/10.6084/m9.figshare.30763049).

## Results

**Fig 3:**
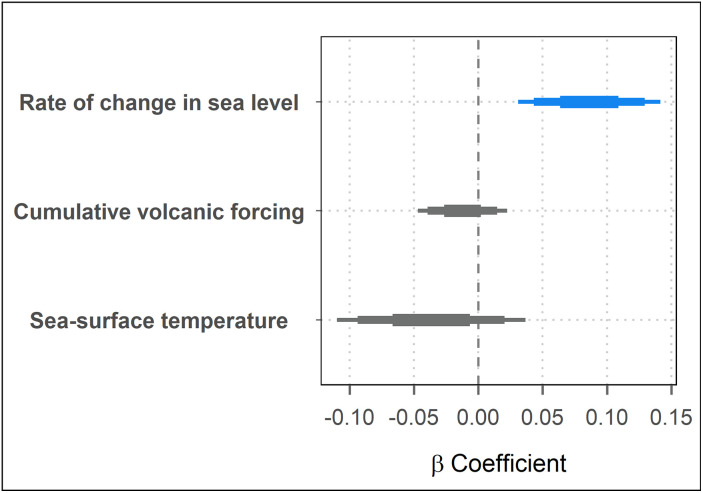
Relationships between three fixed-effect variables and mean coral-reef growth rates (mm/yr) through the Holocene from 291 sites across the Pacific, Indian, and Atlantic Oceans, from 11,700 years before present (yr BP; 1950 Common Era [CE]), through the Holocene. Where the rate of change in sea level is measured in mm/yr, the atmospheric-composition variable cumulative volcanic forcing (W/m^2^) is calculated from the mean stratospheric aerosol optical depth, and SST is the sea-surface temperature (°C). Each coefficient is shown with three horizontal bars representing 90%, 80%, and 50% credible intervals. Coefficients with strong positive effects (≥ 80% credible intervals) are shown in blue. Terms not credibly different from zero (dashed line) are shown in gray.

The results of the Generalized Linear Mixed Model, using the Integrated Nested Laplace Approximation (INLA) approach, showed the rate of change in sea level (mm/yr) as the only variable with a positive relationship with mean coral-reef growth rates over the past 11,700 yr BP (Fig 3). By contrast, cumulative volcanic forcing (W/m^2^) and mean sea-surface temperature (⁰C) showed no credible linear relationships with mean coral-reef growth rates, though both exhibited a tendency toward negative effects (Fig 3; S5 Table in S1 File).

**Fig 4:**
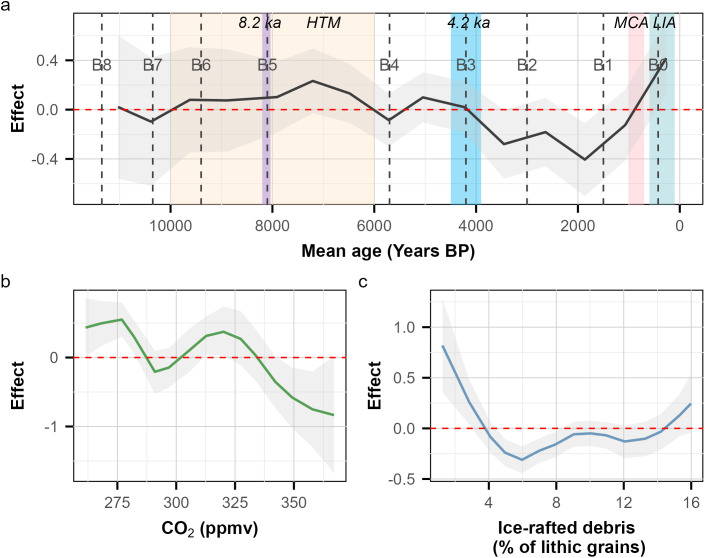
A mixed-modeling approach using second-order random-walk analysis in the Bayesian Integrated Nested Laplace Approximation (INLA) to assess the relationship between mean coral-reef growth rates (mm/yr) and 3 variables through the Holocene from 291 sites across the Pacific, Indian, and Atlantic Oceans, from 11,700 calibrated years before present (yr BP; 1950 Common Era [CE]). **(a)** the effect of mean age of the coral-core samples, where BP is defined as calibrated years before present (1950 CE) is represented by a black line, **(b)** the effect of carbon dioxide (CO_2_) concentrations in parts per million by volume (ppmv) is represented by a green line, **(c)** the effect of stacked percentage of ice-raft debris types (as a proxy for Bond cycles) is represented by a blue line. Positive effects indicate periods where mean growth rates were higher than expected, relative to the overall trend, whereas negative effects indicate mean growth rates were lower than expected. Note that the five major temperature-fluctuation events of the Holocene depicted in (a) are: 1) the Holocene Thermal Maximum (**HTM**) (10,000–6,000 yr BP) [13], shaded in yellow, 2) the **8.2 ka** yr BP cooling event (8,200–8,040 yr BP) [14], shaded in purple, 3) the **4.2 ka** yr BP cooling event (4,500–3,900 yr BP) [15], shaded in blue, 4) the warm Medieval Climate Anomaly (**MCA**) (1,000–700 yr BP) [16], shaded in pink, and 5) the Little Ice Age (**LIA**) (600–100 yr BP) [16], shaded in light green. Also note that the nine **Bond-cycle events** [29] are dashed gray lines, labeled from B0 to B8, where B0 is the most recent cycle.

Coral-reef growth rates (mm/yr) varied throughout the Holocene, with considerable uncertainty in estimates before 8,000 yr BP, partly because of the sparse data available in the early Holocene (Fig 4a). The highest mean growth rates occurred between ~7,000 and ~5,500 yr BP, which coincided with the Holocene Thermal Maximum (Fig 4a). Overall, there was a notable decline in mean growth rates from ~5,000 to ~1,000 yr BP and an increase from ~1,000 yr BP to present (Fig 4a). The random walk Bayesian model applied to atmospheric carbon dioxide (CO_2_) concentrations revealed a bimodal effect on reef growth rates, peaking at ~ 275 and 325 ppmv, followed by a continuous decline (Fig 4b) whereby the effect crossed zero at ~335 ppm and continued to decline. While uncertainty increased for CO₂ concentrations above ~335 ppmv, because of the limited number of samples, the estimated effect remained negative. The Bayesian model also showed that the highest coral-reef growth rates coincided with low percentages of ice-rafted debris of total lithic grains, used as a proxy for Bond cycles (Fig 4c).

Oceans, regions, and localities were included in the model as independent and identically distributed (iid) random effects. Growth rates showed no overall spatial variation across these groups (S9 and S10 Figs in S1 File), although a few localities deviated from zero, with nine showing credible positive and eight showing credible negative effects (S11 Fig in S1 File). The latent spatial field revealed sites with positive and negative deviations from their expected mean, with the highest growth generally observed at sites in the Great Barrier Reef and the lowest growth observed at sites in the Indian Ocean (S12 Fig in S1 File).

**Fig 5:**
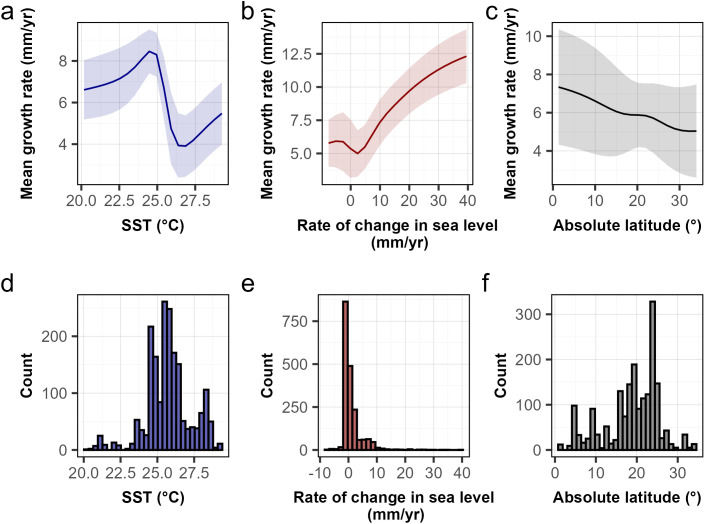
Partial dependency plots resulting from the deep-learning neural-network model show the relationships between mean growth rates (mm/yr) and 3 variables through the Holocene from 291 sites across the Pacific, Indian, and Atlantic Oceans, from 11,700 yr BP. **(a)** sea-surface temperature (SST) (°C) represented by a purple line, **(b)** rate of change in sea level (mm/yr) represented by a maroon line, and **(c)** absolute latitude (°N or °S) over the past 11,700 years represented by a charcoal line. Histograms of the distribution for each variable are shown in panels **(d)** through **(f)**.

The deep-learning neural-network model showed that coral-reef growth (mm/yr) was optimal between 24 and 25 °C throughout the Holocene and declined when sea-surface temperatures were higher than 25 °C (Fig 5a and 5d). Mean coral-reef growth rates increased as the rate of change in sea level increased and began to plateau when the rate of change in sea level was approximately 20 mm/yr (Fig 5b and 5e). Coral reefs at latitudes closer to the Equator grew faster than elsewhere, although there was considerable uncertainty (Fig 5c and 5f).

**Fig 6:**
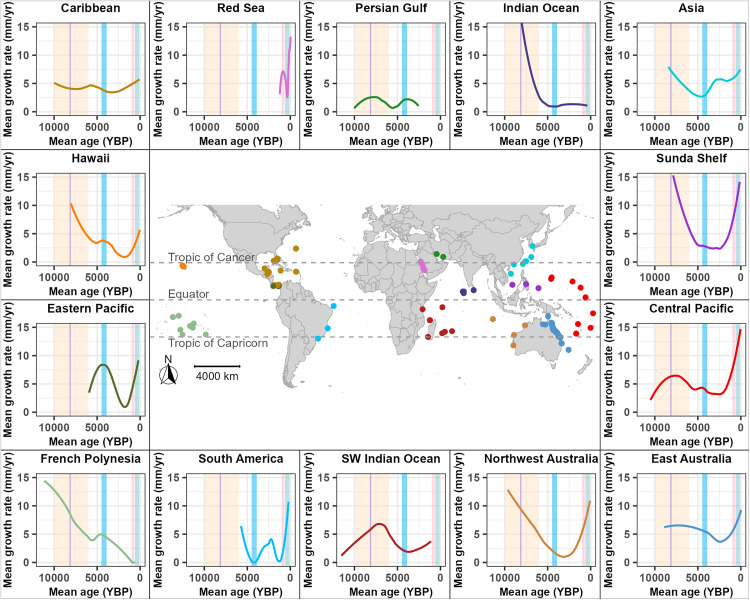
The location of 1,869 samples, dating back 11,700 years before present (YBP; 1950 Common Era [CE]) through the Holocene from 288 sites across the Pacific, Indian, and Atlantic Oceans, with locally estimated scatterplot smoothing (LOESS) mean coral-reef growth rates (mm/yr) (95% confidence interval), grouped in 14 regions globally. The colored LOESS line in each of the 14 regional plots corresponds to the color of sites on the map. The remaining 21 samples in the dataset reside in Barbados and were excluded in this comparison because mean ages were limited to earlier than ~9,000 yr BP (S13 Fig in S1 File). The five major temperature-fluctuation events of the Holocene depicted are: 1) the Holocene Thermal Maximum (10,000–6,000 yr BP) [13], shaded in yellow, 2) the 8.2 ka yr BP cooling event (8,200–8,040 yr BP) [14], shaded in purple, 3) the 4.2 ka yr BP cooling event (4,500–3,900 yr BP) [15], shaded in blue, 4) the warm Medieval Climate Anomaly (1,000–700 yr BP) [16], shaded in pink, and 5) the Little Ice Age (600–100 yr BP) [16], shaded in light green. The map was generated using world polygon data from the *maps* [38] in R [39].

There were geographical differences in mean coral-reef growth rates at distinct periods through the Holocene (Fig 6). For example, coral-reef growth rates in the Caribbean region were relatively stable throughout the Holocene, whereas reef growth rates in the Sunda Shelf, Hawaii, northwestern Australia, French Polynesia, and the northern Indian Ocean all exhibited rapid growth rates during the early Holocene, followed by declines through the Holocene Thermal Maximum. Reef growth rates in Asia were decoupled from those in other regions, exhibiting bimodal growth patterns, with rapid growth in the early Holocene and ~2,000 yr BP and lower rates during other periods. Coral reefs in the central Pacific and eastern Australia also exhibited bimodal growth rates, with peaks at ~8,000 yr BP and ~6,000 yr BP, respectively, followed by declines to low rates at ~2,000 yr BP, and recent increases. Coral-reef growth in the southwestern Indian Ocean peaked in the mid-Holocene but reached an earlier low at ~4,000 yr BP than reefs in eastern Australia and the Central Pacific. Coral reefs in the Red Sea exhibited relatively high, albeit variable, growth rates, although temporal coverage was sparse (Fig 6). By contrast, coral-reef growth in the Persian (Arabian) Gulf was low throughout the Holocene. The eastern Pacific displayed a different pattern than the other regions, exhibiting a mid-Holocene growth pulse followed by a near-complete growth hiatus lasting 2,500 years, beginning around ~4,200 yr BP. Coral-reef growth rates in South America were highly variable and noticeably rapid over the past 1,000 years. Similarly, coral-reef growth in the Sunda Shelf, northwestern Australia, and Hawaii also showed rapid increases over the past 3,000 years.

Coral-reef growth rates showed similar trends across coral-reef zones (i.e., fringing reefs, reef crests, back reefs, and fore reefs). Across all four coral-reef zones (i.e., habitats), the highest rates of coral-reef growth (mm/yr) occurred in the early Holocene and generally declined over time (S14a Fig in S1 File). In the past 2,500 yr BP, the rates of coral-reef growth increased on the fore reefs and back reefs, whereas the fringing reefs did not show this recent increase. Overall, the reef crest exhibited the highest coral-reef growth rates, but also showed the largest decline during the Holocene Thermal Maximum 10,000–6,000 yr BP. By contrast, the fore reefs and back reefs maintained more consistent but lower coral-reef growth rates than the reef crests through the Holocene.

## Discussion

Overall, our results showed considerable regional differences in coral-reef growth rates (mm/yr) throughout the Holocene, with the fastest overall growth rates occurring when sea-surface temperatures were ~25 °C during the Holocene Thermal Maximum, between ~7,000 and 5,500 yr BP. Rapid rates of coral-reef growth occurred after the rapid increase in sea level through the early Holocene transgression period. The positive relationship between coral-reef growth and rates of sea-level change agrees with Montaggioni [3], who reviewed data from 684 subsurface cores spanning the Holocene and found that reef growth in the Indo-Pacific Ocean followed two main phases. The first phase, the so-called transgressive phase, followed deglaciation around 16.5 ka yr BP, with sea level increasing rapidly around 14 ka yr BP and then slowing around ~7 ka yr BP when Earth experienced sea-level highstands. During this period, coral reefs underwent significant depositional processes, resulting in aggradation and shallowing toward sea level [3]. This transgressive phase was followed by a still-stand phase after sea level had stabilized. The still-stand phase was characterized by lateral reef growth and back-reef infilling. Most of our study data aligns with the still-stand phase.

While orbital forcing cycles are longer than the temporal extent of this study, during the early Holocene, precessional forcing was low, and reef growth rates (mm/yr) exceeded the Holocene average (Fig 1). Low precessional forcing minimized interannual seasonality between hemispheres through an even solar insolation (W/m^2^) distribution across latitudes, stabilizing the climate. As a result, stable sea-surface temperatures likely led to optimal coral [83] and reef growth when temperatures were ~25 °C. Precessional forcing increased after the Holocene Thermal Maximum, and reef growth rates declined. Increased precessional forcing shifted the distribution of solar insolation and intensified interannual seasonality, leading to an increase in the variability of the El Niño-Southern Oscillation [84]. Consequently, sea-surface temperature anomalies, upwelling dynamics, and altered precipitation and runoff patterns would have disrupted favorable reef-building conditions, especially in areas sensitive to thermal stress and abrupt environmental change. Except in Asia, global declines in reef growth rates were evident immediately following the Holocene Thermal Maximum period. However, around 3,000 yr BP, reef growth rates increased in most regions, coinciding with a plateau in precessional forcing.

During climatic instability, around 4,200 yr BP, reef growth in the eastern Pacific Ocean ceased for approximately 2,600 years, an event purportedly linked to increased El Niño variability [22]. A Bond-cycle event also coincided with the onset of the 4.2 ka yr BP cooling event [85,86]. Bond cycles are recognized as the percentage of ice-rafted debris found in North Atlantic sediment cores [11,29]. These ice-rafted debris indices represent the changes in North Atlantic drift ice, detached from Greenland, cooling the ocean, and disrupting oceanic circulation [11]. Such disruptions shifted heat accumulation toward the tropics, driving a southward shift in the Intertropical Convergence Zone. During this 4.2 ka yr BP cooling event, coral-reef growth declined in the eastern Pacific Ocean [22]. By contrast, during the 4.2 ka yr BP cooling event, coral reefs in the southwestern Indian Ocean, parts of South America, and northwestern Australia experienced low but continuous reef growth [87]. Yet, during the same period, coral-reef growth in the South China Sea remained stable [88]. Regional variation in coral-reef growth trends suggests that the cooling during the 4.2 ka yr BP cooling event was likely amplified in some regions of the oceans, such as the eastern Pacific Ocean, that were heavily influenced by North Atlantic Ocean cooling.

Our results also showed a negative relationship between mean rates of reef growth (mm/yr) and latitude (°N or °S), suggesting that coral reefs between latitudes 10° and the Equator grew more rapidly than those at other latitudes. These differences were likely related to differential irradiance, as sunlight strikes Earth at an oblique angle at high latitudes, spreading energy over a larger surface area than at low latitudes, thereby reducing reef growth capacity at high latitudes [34]. Previously, Montaggioni [3] noted no major variation in reef growth rates across latitudes, except in the Hawaiian archipelago. By contrast, our results show a moderate relationship with latitude.

Overall, coral-reef growth rates in Asia, the eastern Pacific, and the northern Indian Ocean were distinctly different from those in all other regions and varied among themselves. By contrast, some regions, such as Hawaii, northwestern Australia, and the Sunda Shelf, exhibited similar reef growth trajectories throughout the Holocene, despite being geographically distant from each other. The central Pacific, eastern Australia, and southwestern Indian Ocean also had similar trajectories, although their inflection points differed. The latent spatial effects, which display geographical results that were not captured by our data, show positive deviations from the expected growth rates at Papeete (French Polynesia), Yanbu and Al-Lith (Saudi Arabia), Hayman Island (Whitsundays, Australia), Pandora Reef (Queensland, Australia), Middle Reef (Magnetic Island, Australia), Funafuti Atoll (Tuvalu), and Yoron Island (Japan). There were negative deviations from expected growth rates evident at Qeshm Island [Persian (Arabian) Gulf], Boat Urunu Faru (Maldives), Wellington Point (Moreton Bay, Australia), Wedge Island (Keppel Islands, Australia), Bermuda North Lagoon, Abrolhos (Brazil), Karagan Lagoon (Sri Lanka), and Yongle Atoll (China).

Reef growth can also differ among reef habitats and coral assemblages. Although the present study did not examine differences in coral assemblages, previous studies have suggested that the composition of the reef framework builders influenced reef growth rates [3,33,89–91]. Branching coral assemblages were long thought to be associated with the fastest rates of reef growth, whereas massive and encrusting coral assemblages were thought to be associated with the slowest rates of reef growth. However, recently, Roff et al. [92] found that accretion rates on adjacent Great Barrier Reef slopes were decoupled from assemblage composition. While massive *Porites* corals are generally considered slow-growing species and branching *Acropora* corals are fast-growing species, Roff [92] showed that coral composition played no role in the overall rate of reef growth for over 750 years. Our results, however, did show that coral reefs exposed to high-wave energy (i.e., exposed reef crests) grew faster than reefs exposed to low-wave energy (i.e., back reefs and fringing reefs) (S14 Fig in S1 File). These results contrast with those of Montaggioni [3], who reported that high-energy coral reefs did not exceed 12 mm/yr in vertical growth, whereas leeward, low-energy reefs grew at a rate of more than 20 mm/yr in vertical growth. Interestingly, our results align with previous studies on contemporary Pacific Ocean coral reefs, which showed that leeward, nearshore reefs have a lower capacity for carbonate production and reef growth than exposed reefs, and therefore do not grow as rapidly as wave-exposed reefs [93]. High carbonate production and reef growth on wave-exposed reefs are likely driven by strong-water movement, which enhances mass transfer and thereby facilitates coral growth [94]. In a modern context, contemporary nearshore leeward coral reefs are also located near human populations, where local pollution and land-use change exacerbate reductions in growth capacity. Therefore, while some nearshore coral reefs may have the capacity to grow rapidly under optimal environmental conditions [3], nearshore reefs have a lower innate capacity to grow, compared with high-energy exposed reefs, and are particularly vulnerable to present day human disturbances.

Reef growth peaked around 24–25 °C through the Holocene and declined once atmospheric CO₂ exceeded ~335 ppm. These results suggest that optimal reef growth occurs at temperatures between 24 and 25 °C and declines at higher temperatures. Indeed, ocean temperatures three to four degrees higher than this optimum, depending on the ocean, have led to recent coral bleaching and mortality [95], which caused reductions in carbonate production [93,96]. Mesocosm studies simulating future ocean temperatures and ocean acidification, at even higher atmospheric CO₂ than today, show direct negative effects on reef calcification and the loss of carbonate framework [97]. However, several studies have shown that the onset of coral bleaching has recently occurred at higher temperatures than in past decades [98], with the global median coral-bleaching temperature changing from ~28 °C during 1980–1999 to ~29 °C during 2010–2020 [99]. These results suggest that corals are capable of adjusting to a higher thermal threshold, although such adjustments are limited by the rate of increase in ocean temperatures. Clearly, the increasing frequency of marine heatwaves and lethal and sub-lethal bleaching events at higher ocean temperatures will reduce the capacity of reefs to grow [100].

While our study was instructive, some notable limitations inherent to large-scale paleo-studies are also present in our study. Wide-ranging core-segment lengths can incorporate both short-term and long-term resolutions. Incorporating core-segment length as a second-order random walk in our models helps to smooth these differences, but the effective temporal resolution of the resulting estimates remains constrained by the original sampling interval of each core segment. Consequently, short-term variability may be underrepresented in long-core segments, whereas long-term trends may be overemphasized in short-core segments. Secondly, because some environmental predictor variables were unavailable at the spatial and temporal resolutions of our dataset, the variables were linearly interpolated across time steps or latitudes. Such interpolations can add bias. Thirdly, historical detailed data on paleo-bioerosion, sedimentation rates, and accretion depth — key controls of reef growth [93,101] — are limited in coverage in space and time and are therefore excluded from the model. Despite these limitations, this study provides the most comprehensive global analysis of coral-reef growth to date, synthesizing 1,890 samples from 291 geographically distributed sites spanning the past 11,700 yr BP since the last ice age, throughout the Holocene. Building on geographically focused studies [15,23,102,103], this analysis enables large-scale, long-term, and interregional comparisons of coral-reef growth across diverse environmental conditions. This study incorporates a wide array of environmental and climate variables reconstructed from multi-proxy datasets to identify long-term drivers and explore non-linear relationships influencing coral-reef growth—using an advanced statistical modeling approach with a spatial mesh, we capture these relationships and their spatial dependencies.

In conclusion, we found that the optimal sea-surface temperature for coral-reef growth (mm/yr) throughout the Holocene was between 24 and 25 °C, that reef growth declined at atmospheric carbon dioxide (CO_2_) concentrations of > 335 parts per million by volume (ppmv), and at latitudes further from the Equator. Modern levels of CO_2_ are reported at 425 ppmv, strongly suggesting that these high levels of CO_2_, associated with high emissions of greenhouse gases, are less than optimal for contemporary coral-reef growth. In addition, the high intensity and frequency of modern marine heatwaves [104] are causing coral bleaching and mortality, which hinder coral-reef growth [93,96]. There is concern for the future of coral reefs, as marine heatwaves are likely to intensify, and sea levels are expected to continue rising. The responses of coral reefs to past changes in environmental conditions throughout the Holocene makes it clear that the responses of present-day coral reefs to modern disturbances will vary geographically, as environmental conditions differ across regions. However, unless greenhouse-gas emissions are reduced and rapid adaptation occurs, coral-reef growth will decline as ocean temperatures increase.

## Supporting information

S1 File Fourteen supporting figures and five supporting tables. (PDF)
