# Supplementary material for: Growth rates of coral reefs peaked at 25 °C through the Holocene
Source: PLoS One. 2026 Mar 11;21(3):e0342527. doi: 10.1371/journal.pone.0342527 (PMC12978502; doi:10.1371/journal.pone.0342527)
Supplement: S1 File — (PDF) [file pone.0342527.s001.pdf]

Supporting information

S1 File. Fourteen supporting figures and five supporting tables.

This is a supporting document for the paper entitled “Growth rates of coral reefs peaked at 25 ºC through the Holocene”, by Tonya Macedo and Robert van Woesik.

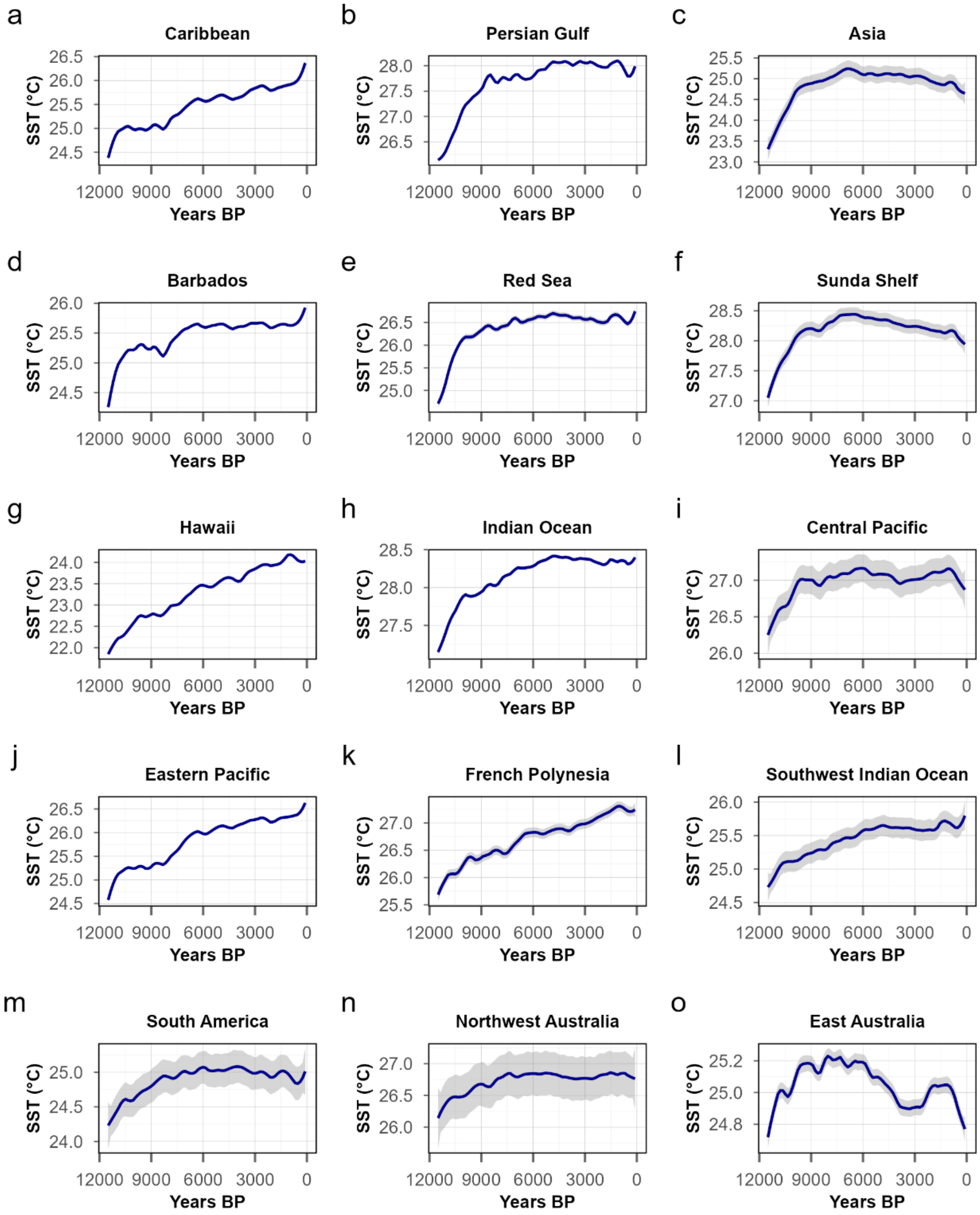

S1 Fig. Locally estimated scatterplot smoothing (LOESS) plot for sea-surface temperature (SST) reconstructions for 15 regions mapped in Figure 2 over the Holocene in years before present (Years BP; 1950 Common Era [CE]). The dark blue line represents the LOESS-smoothed SST, and the shaded gray area indicates the 95% confidence interval. Each panel shows sea-surface temperature for a single region with independent y-axis scales. Data were from the gridded Last Glacial Maximum Reanalysis (LGMR) dataset available via the National Oceanic and Atmospheric Administration (NOAA) Paleoclimatology Data Archive from Osman et al. [1]

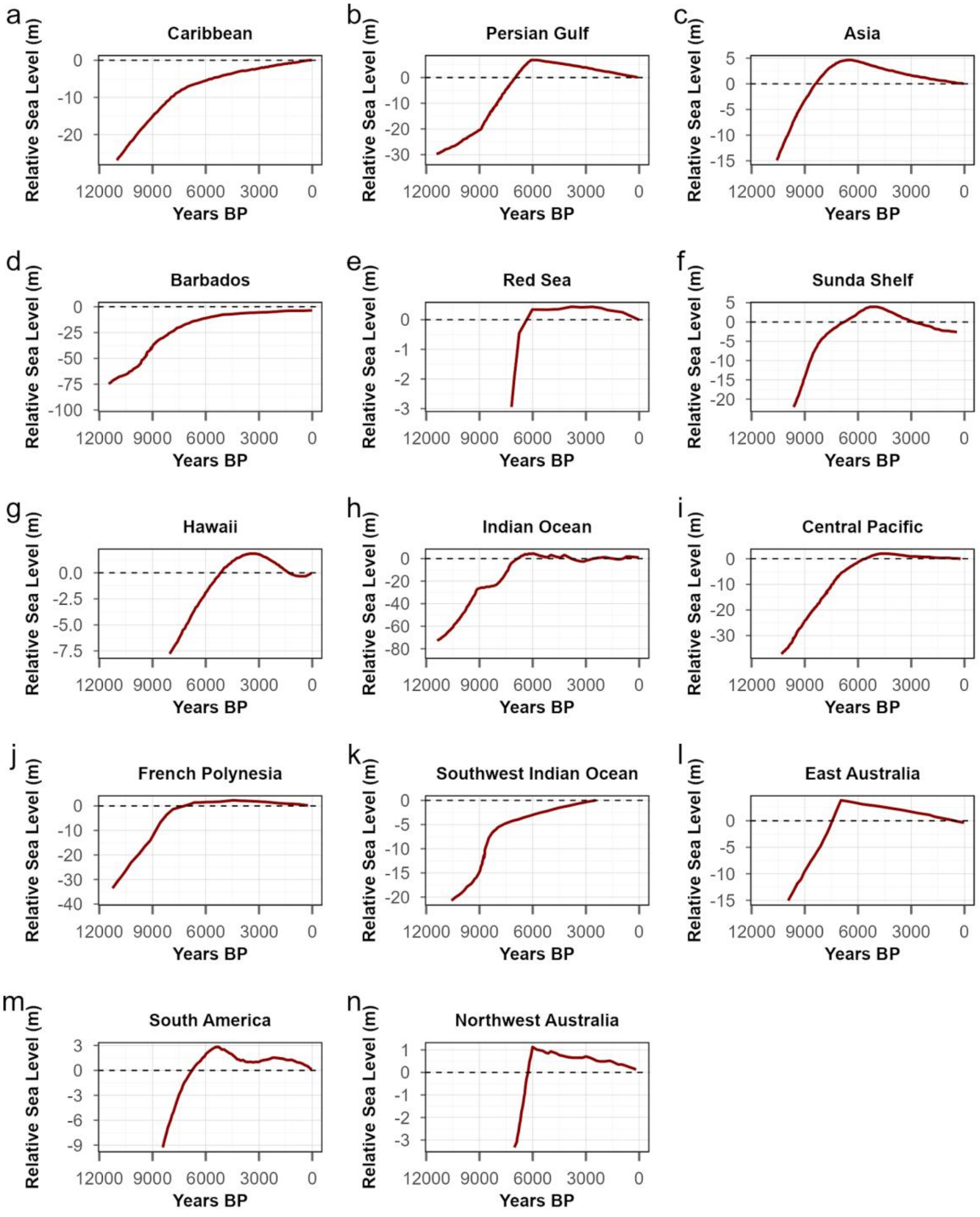

S2 Fig. Relative sea-level reconstructions for 14 regions mapped in Fig. 2 over the Holocene in years before present (Years BP; 1950 Common Era [CE]), where the eastern Pacific Ocean region is grouped with the Caribbean because of sparse sea-level data for that region. Each panel shows relative sea level for a single sea-level region with independent y-axis scales. Data were compiled from published regional sea-level reconstructions, (a) [2]; (b) [3]; (c) [4]; (d) [5]; (e) [6]; (f) [7]; (g) [8]; (h) [9]; (i) [10]; (j) [11]; (k) [12]; (l); [13]; (m) [14]; (n) [15].

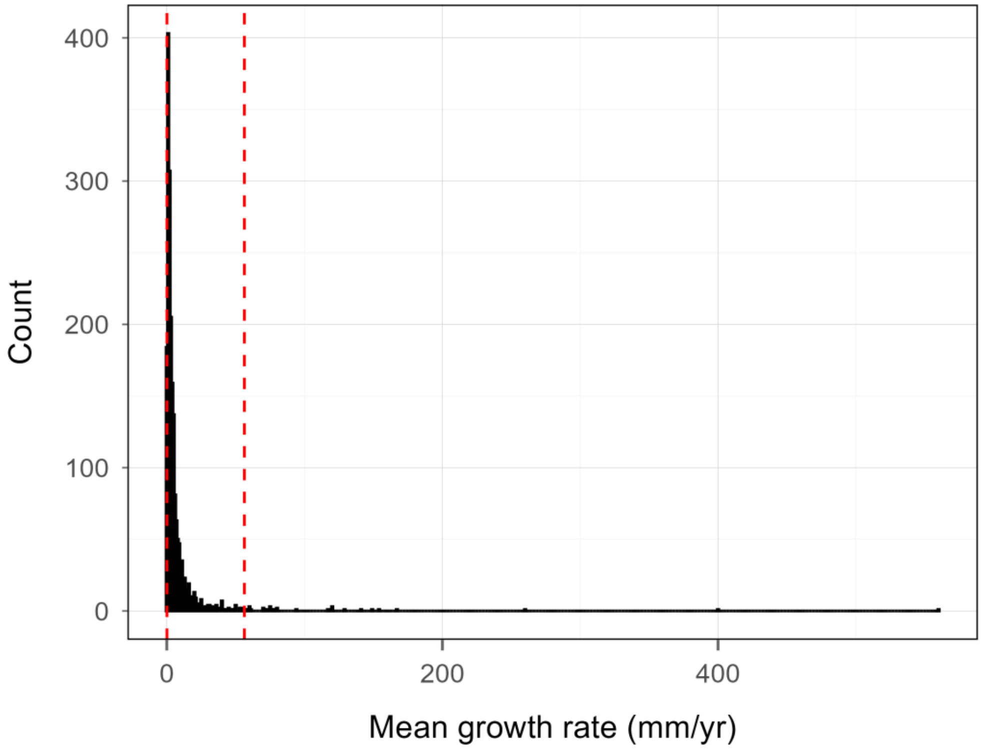

S3 Fig. Raw data histogram of mean coral-reef growth rates (mm/y) for 1,950 samples from 291 sites across the Pacific, Indian, and Atlantic Oceans, from 11,700 years before present (BP; 1950 Common Era [CE]), through the Holocene. The dashed red lines indicate the 97th two-tailed percentile of the distribution. Samples between this threshold were retained for analysis (1,890 samples), whereas those outside were considered outliers and excluded as biologically unrealistic or temporally biased. The range of mean coral-reef growth rate is ~ 0.11 to 53.6 mm/yr.

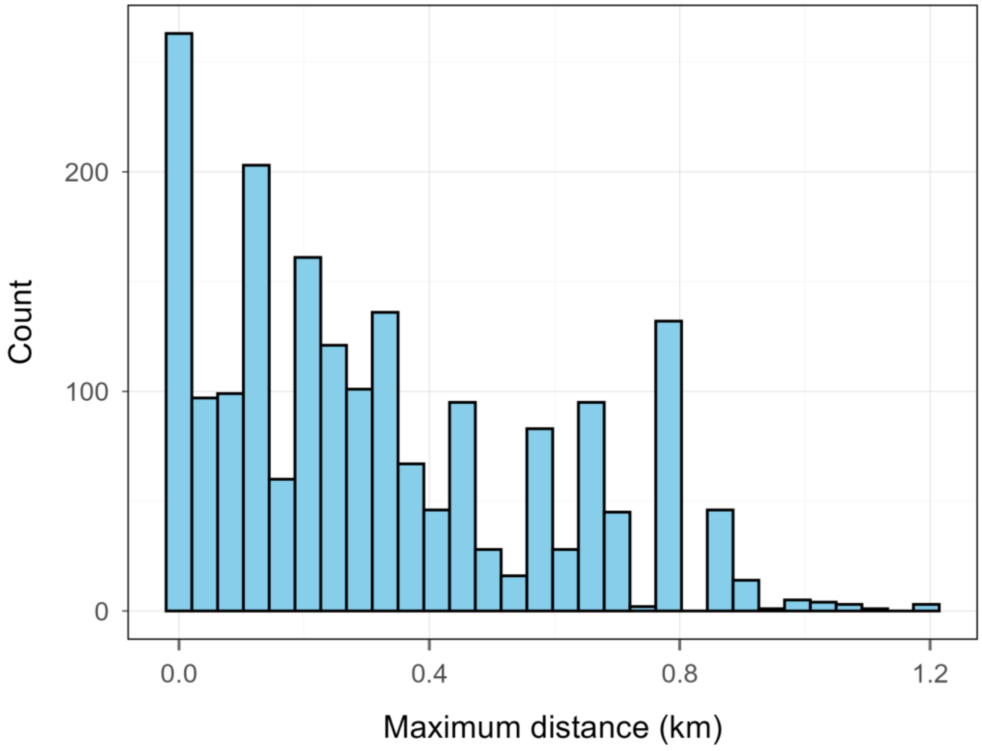

S4 Fig. Histogram of maximum geodesic distances in kilometers between modern coordinates and the paleo coordinate estimates derived from any of the five models (Merdith 2021, TorsvikCocks 2017, Paleomap, Matthews 2016, and Golonka) using *paleorotate* [16] in R. The coordinates were derived from 1,890 samples at 291 sites across the Pacific, Indian, and Atlantic Oceans, from 11,700 years before present (BP; 1950 Common Era [CE]), through the Holocene.

A

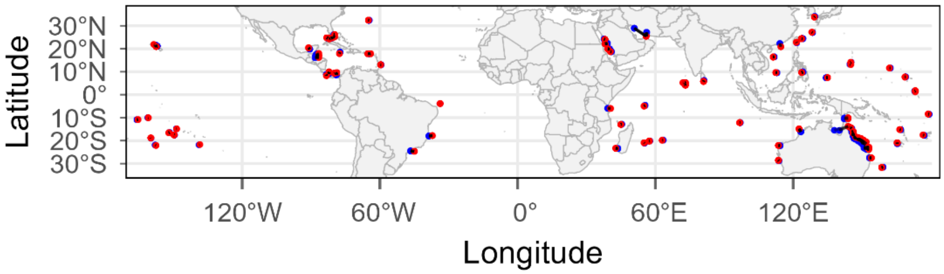

B

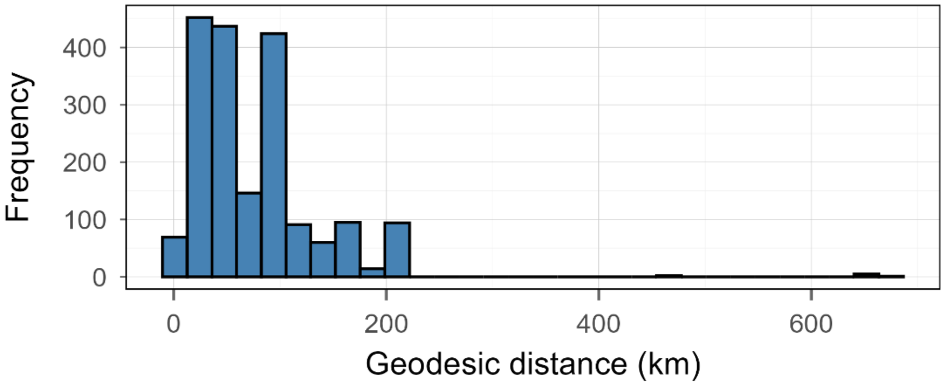

S5 Fig. (a) Map of sample coordinates in blue, and the nearest valid cell containing a value for sea-surface temperature coordinates in red. (b) A histogram of the distribution of geodesic distance in kilometers (km) between the sample coordinates and the nearest valid cell. The coordinates were derived from 1,890 samples at 291 sites across the Pacific, Indian, and Atlantic Oceans, from 11,700 years before present (BP; 1950 Common Era [CE]), through the Holocene. The basemap was created using world polygon data from Natural Earth (public domain; http://www.naturalearthdata.com) via *rnaturalearth* in R [17].

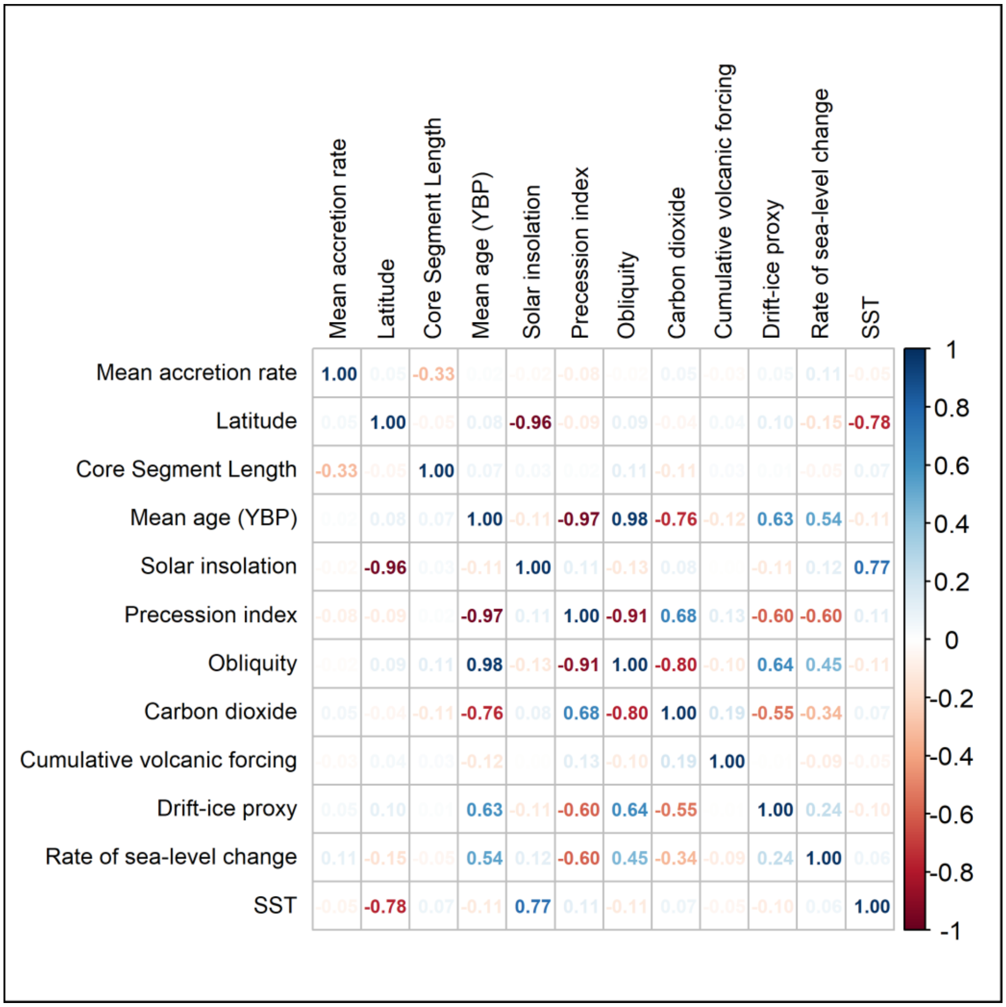

S6 Fig. Correlation plot comparison of the environmental and climatic variables with mean coral-reef growth rate (mm/yr). Where latitude is the absolute latitude (ºN or ºS), core segment length is in mm, mean age is in years before present (YBP; 1950 Common Era [CE]), solar insolation is spatio-temporally adjusted total solar insolation (W/m^2), precession index is calculated as *eccentricity* × *sin(ω)*, obliquity is Earth's tilt in degrees, CO_2 is carbon dioxide (ppmv), cumulative volcanic forcing is the 20-year rolling mean volcanic forcing (W/m^2), drift-ice proxy is the combined percentage of ice-rafted debris (hematite-stained grains, detrital carbonate, and Icelandic volcanic glass) of total lithic grains, rate of change in sea level is the mean rate of change in meters, and sea-surface temperature is the mean in °C. The data were from 1,890 samples at 291 sites across the Pacific, Indian, and Atlantic Oceans, from 11,700 years before present (BP; 1950 CE) through the Holocene.

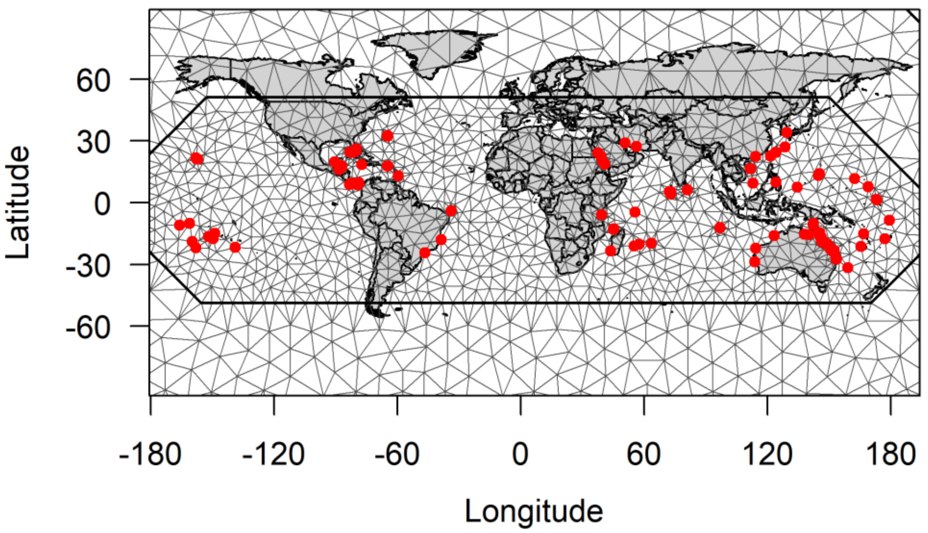

S7 Fig. Spatial mesh used for the Integrated Nested Laplace Approximation (INLA) Stochastic Partial Differential Equation (SPDE) model created by the INLA [18] package in R. The triangulated mesh (black lines) consists of 3,391 nodes, providing fine spatial resolution across the study domain. Red points indicate the locations of 1,890 observed samples used to construct the mesh and fit the spatial random field. The data were from 291 sites across the Pacific, Indian, and Atlantic Oceans, from 11,700 years before present (BP; 1950 CE) through the Holocene. The basemap was generated using the *maps* package [19] in R.

S1 Table. Binning groups for variables included as second-order random walks in the general linear mixed model (GLMM) in Integrated Nested Laplace Approximation (INLA) using 4 environmental variables. The data were from 1,890 samples at 291 sites across the Pacific, Indian, and Atlantic Oceans, from 11,700 years before present (BP; 1950 Common Era [CE]), encompassing the Holocene.

Variable	Unit	Number of Bins	Range
Mean age	Years before present	15	-40.5 - 11,520.0
Core segment length	Years	10	1 - 8,786
CO_2	Parts per million by volume	15	257.77 - 368.02
Ice-rafted debris	Percent of lithic grains	15	1.25 - 16.78

S2 Table. Results from generalized linear mixed models comparing three correlated predictor variables: sea surface temperature (ºC), solar insolation (W/m^2), and latitude (ºN or ºS). Model fit was assessed using the Watanabe-Akaike information criterion (WAIC), the deviance information criterion (DIC), and the mean conditional predictive ordinate (CPO Mean). Random effect terms (RW) correspond to variables modeled as second-order random walks, with the number following each RW term indicating the number of bins used. Independent and identically distributed (iid) effects are reported in the iid Terms column. The data were from 1,890 samples at 291 sites across the Pacific, Indian, and Atlantic Oceans, from 11,700 years before present (BP; 1950 Common Era [CE]), through the Holocene.

Model Name	WAIC	DIC	CPO Mean	Fixed Effects	RW Terms	iid Terms
model1	9143.781	9099.788	0.176096	Intercept, SST, Cumulative volcanic forcing, Rate of change in sea level	age_15, core_length_10, CO2_15, drift_15	Ocean, Region, Locality, Core ID
model3	9145.422	9101.517	0.17632	Intercept, Absolute latitude, Cumulative volcanic forcing, Rate of change in sea level	age_15, core_length_10, CO2_15, drift_15	Ocean, Region, Locality, Core ID
model2	9151.294	9108.545	0.174311	Intercept, Solar insolation, Cumulative volcanic forcing, Rate of change in sea level	age_15, core_length_10, CO2_15, drift_15	Ocean, Region, Locality, Core ID

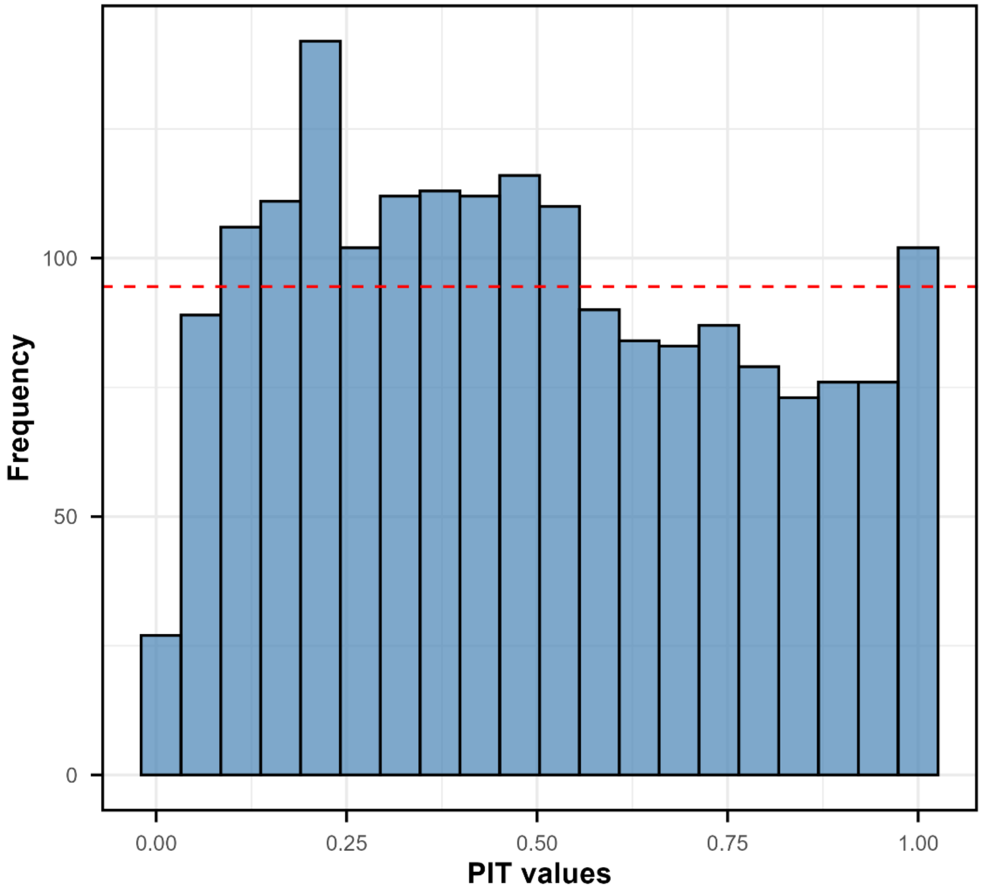

S8 Fig. Probability integral transform (PIT) histogram of the general linear mixed model residuals, ranging from 0.0 to 1.0. PIT values near 0.0 indicate the model underestimates the observation, while values near 1.0 indicate it overestimates the observation. The red dashed line marks the expected height for a perfectly uniform distribution. The data were from 1,890 samples at 291 sites across the Pacific, Indian, and Atlantic Oceans, from 11,700 years before present (BP; 1950 Common Era [CE]) through the Holocene.

S3 Table. Model architecture for six deep-learning models used for comparison, including the number of hidden layers, epochs (training iterations), stopping rounds (early stopping), activation function, input and hidden dropout rates (regularization), and n-fold cross-validation. Each configuration was applied to evaluate nonlinear relationships between mean coral-reef growth rates and three predictor variables: sea-surface temperature (ºC), rate of sea-level change (mm/yr), and absolute latitude (ºN or ºS). The data were from 1,890 samples at 291 sites across the Pacific, Indian, and Atlantic Oceans, from 11,700 years before present (BP; 1950 Common Era [CE]) through the Holocene.

Model ID	Hidden layers	Epochs	Stopping rounds	Activation	Input dropout	Hidden dropout	N folds
dl_fit1	NA	1	0	Tanh	0	NA	0
dl_fit2	10-10	20	3	Tanh	0	NA	0
dl_fit3	20-10	100	5	Tanh	0	NA	0
dl_fit4	15-15	100	5	Tanh	0	NA	0
dl_fit5	15-10	100	5	Tanh	0	NA	5
dl_fit6	15-10	200	5	Tanh With Dropout	0.1	0.2-0.2	5

S4 Table. Performance comparison of the 10 top-ranked models, ranked by root mean square error (RMSE), across 30 runs of six deep learning model configurations. Additional metrics include mean absolute error (MAE), mean square error (MSE), and the coefficient of determination (R^2). Model dl_fit3, run 11 is in bold, identifying the selected model used to assess nonlinear relationships between reef growth, sea-surface temperature (ºC), rate of change in sea level (mm/yr), and absolute latitude (ºN or ºS). The data were from 1,890 samples at 291 sites across the Pacific, Indian, and Atlantic Oceans, from 11,700 years before present (BP; 1950 Common Era [CE]) through the Holocene.

Model ID	Run Number	RMSE	MAE	MSE	R^2	Rank
**dl_fit3**	**11**	**7.339424**	**4.660292**	**53.86714**	**0.042404**	**1**
dl_fit5	20	7.341555	4.661014	53.89843	0.041848	2
dl_fit5	16	7.343241	4.54624	53.92318	0.041408	3
dl_fit4	16	7.343517	4.878209	53.92724	0.041336	4
dl_fit6	2	7.34471	4.822649	53.94477	0.041024	5
dl_fit3	7	7.349828	4.787113	54.01997	0.039687	6
dl_fit5	24	7.350449	4.827312	54.0291	0.039525	7
dl_fit2	4	7.354499	4.584341	54.08866	0.038466	8
dl_fit6	25	7.354653	4.578363	54.09092	0.038426	9
dl_fit4	30	7.357297	4.598326	54.12982	0.037735	10

S5 Table. Summary of 4 fixed effects from the generalized linear mixed model (GLMM) fit with Integrated Nested Laplace Approximation (INLA). For each effect, the table reports the posterior mean, standard deviation, and the 2.5% and 97.5% quantiles of the posterior distribution, corresponding to the bounds of the 95% credible interval. The data were from 1,890 samples at 291 sites across the Pacific, Indian, and Atlantic Oceans, from 11,700 years before present (BP; 1950 Common Era [CE]) through the Holocene.

Effect	Mean	Standard deviation	0.025 quantile	0.975 quantile
Intercept	-0.4241	0.195501	-0.81353	-0.04598
SST	-0.03662	0.044541	-0.12395	0.050835
Cumulative volcanic forcing	-0.01234	0.021087	-0.05369	0.029016
Rate of change in sea level	0.0863	0.033591	0.02043	0.152173

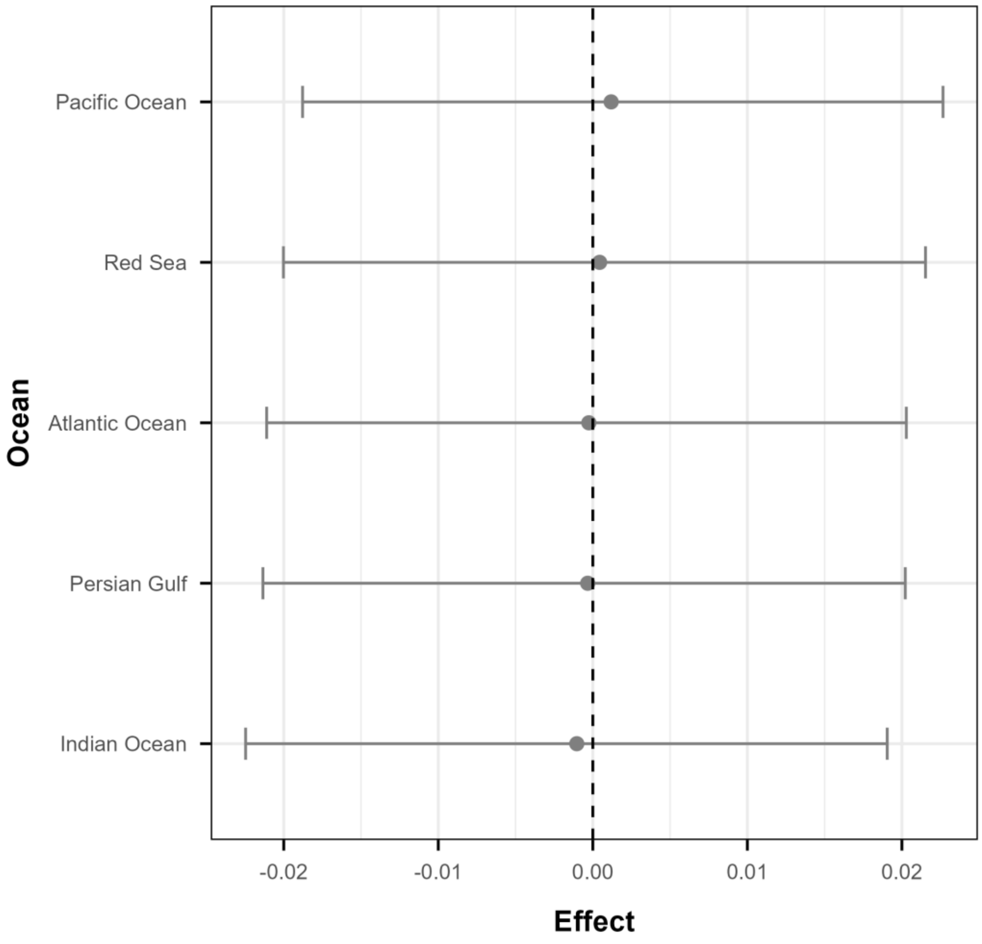

S9 Fig. The estimated mean effect of each ocean on coral-reef growth (mm/yr) with 95% credible intervals. The zero line indicates no effect, and oceans are ordered to highlight relative differences in their influence on the modeled outcome. The data were from 1,890 samples at 291 sites across the Pacific, Indian, and Atlantic Oceans, from 11,700 years before present (BP; 1950 Common Era [CE]) through the Holocene.

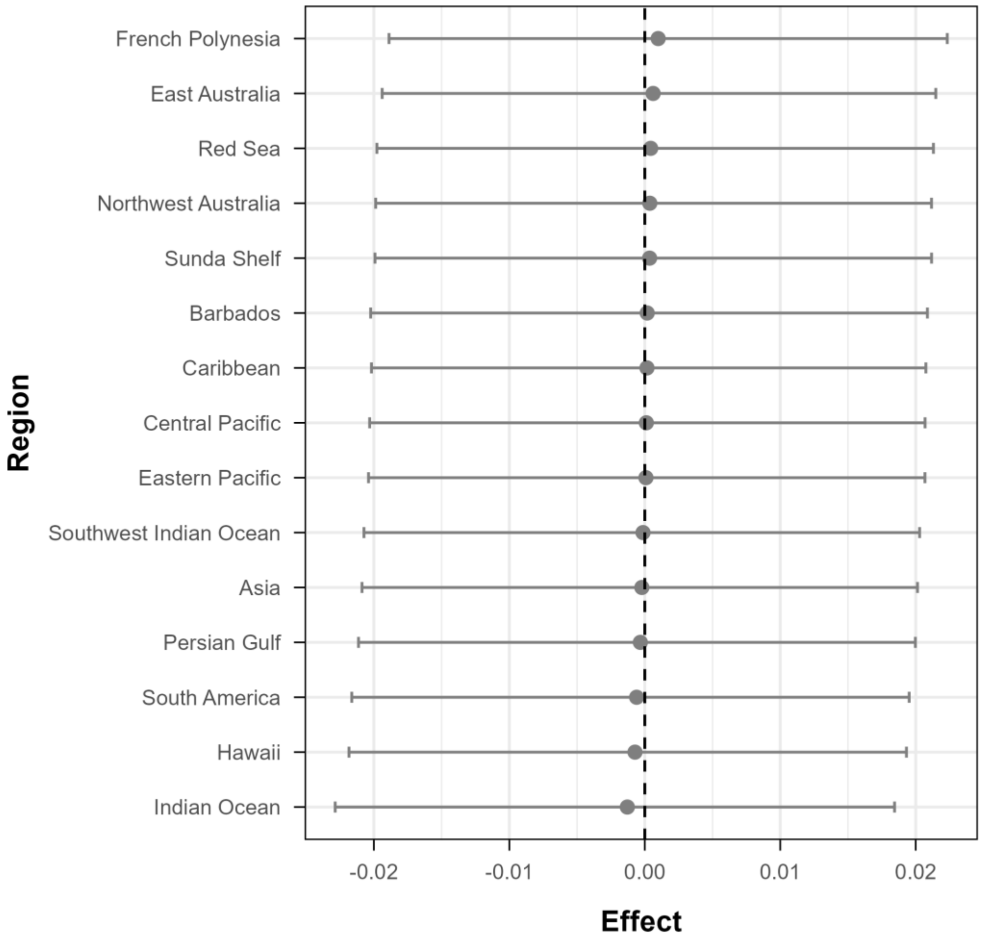

S10 Fig. The estimated mean effect of each of 15 regions on coral-reef growth (mm/yr) with 95% credible intervals. The zero line indicates no effect, and regions are ordered to highlight relative differences in their influence on the modeled outcome. The data were from 1,890 samples at 291 sites across the Pacific, Indian, and Atlantic Oceans, from 11,700 years before present (BP; 1950 Common Era [CE]), through the Holocene.

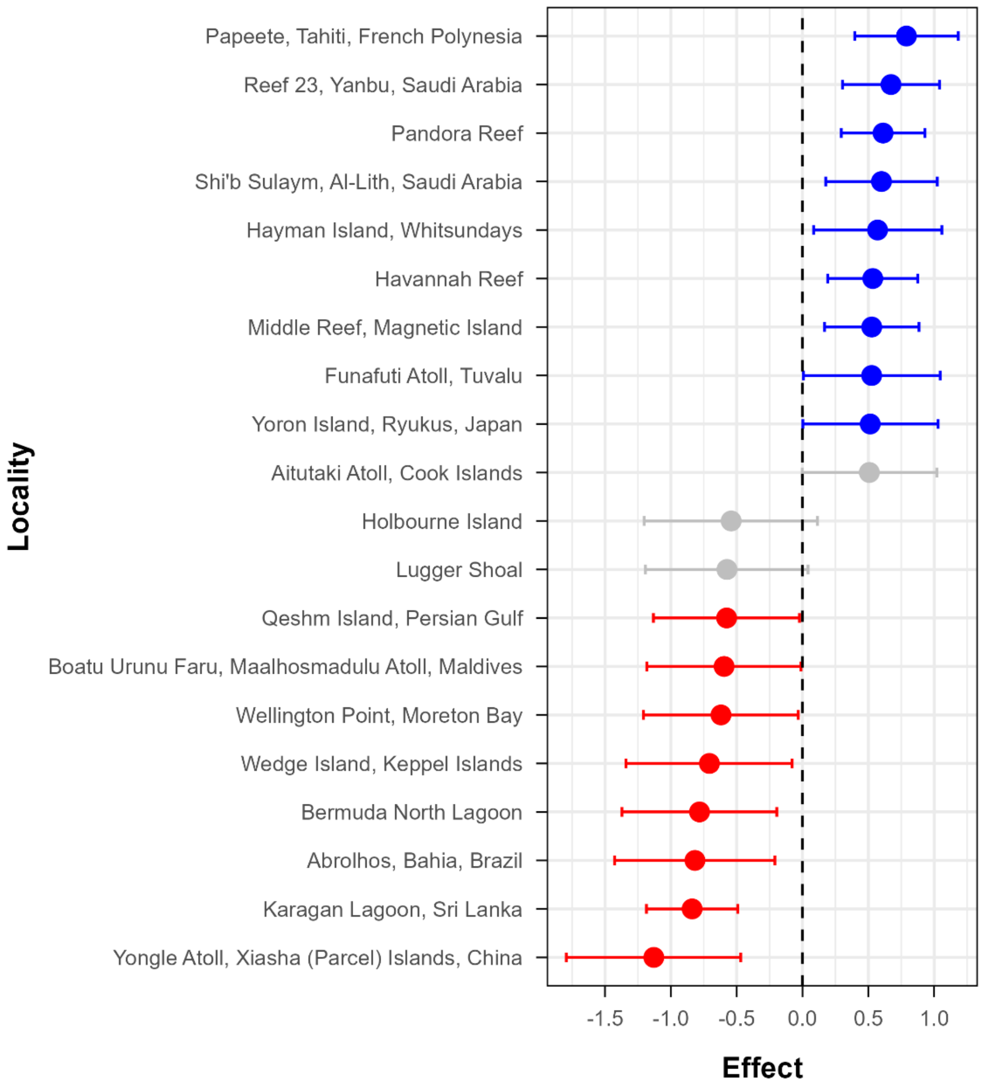

S11 Fig. The estimated mean effect of the highest 10 and lowest 10 localities influencing coral-reef growth (mm/yr). The error bars show the 95% credible intervals. The zero line indicates no effect, and regions are ordered to highlight relative differences in their influence on the modeled outcome. Regions in blue represent the entire credible interval above 0. Regions in red represent the entire credible interval below 0. The data were from 1,890 samples at 291 sites across the Pacific, Indian, and Atlantic Oceans, from 11,700 years before present (BP; 1950 Common Era [CE]) through the Holocene.

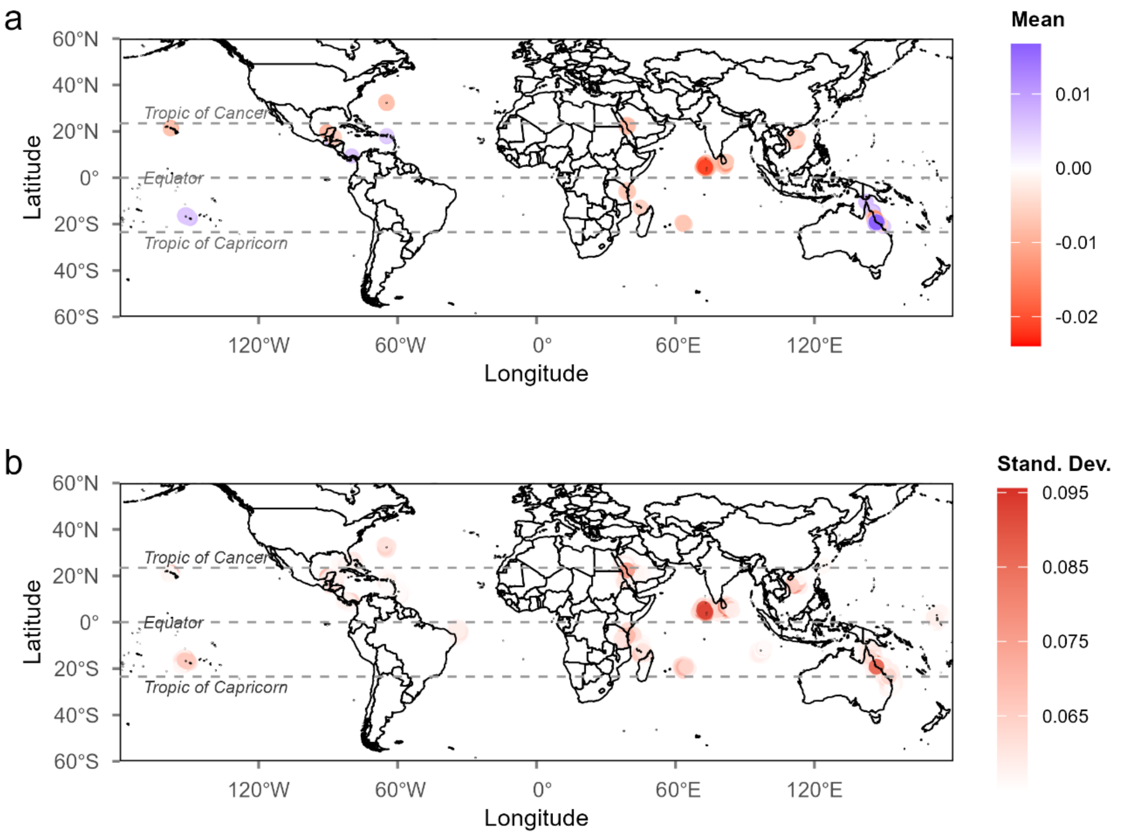

S12 Fig. Spatial projection of the latent random field estimated using Integrated Nested Laplace Approximation (INLA). (a) the posterior mean of the field, (b) the posterior standard deviation (Stand. Dev.). Dashed gray lines indicate the Tropic of Cancer (23.4° N), Equator (0°), and Tropic of Capricorn (23.4° S). Points are colored on a blue–white–red diverging scale (mean-centered at zero), with white indicating values near zero. Legends on the right indicate the color scale for each panel. The data were from 1,890 samples at 291 sites across the Pacific, Indian, and Atlantic Oceans, from 11,700 years before present (BP; 1950 Common Era [CE]) through the Holocene. The basemap was created using world polygon data from Natural Earth (public domain; http://www.naturalearthdata.com) via *rnaturalearth* in R [17].

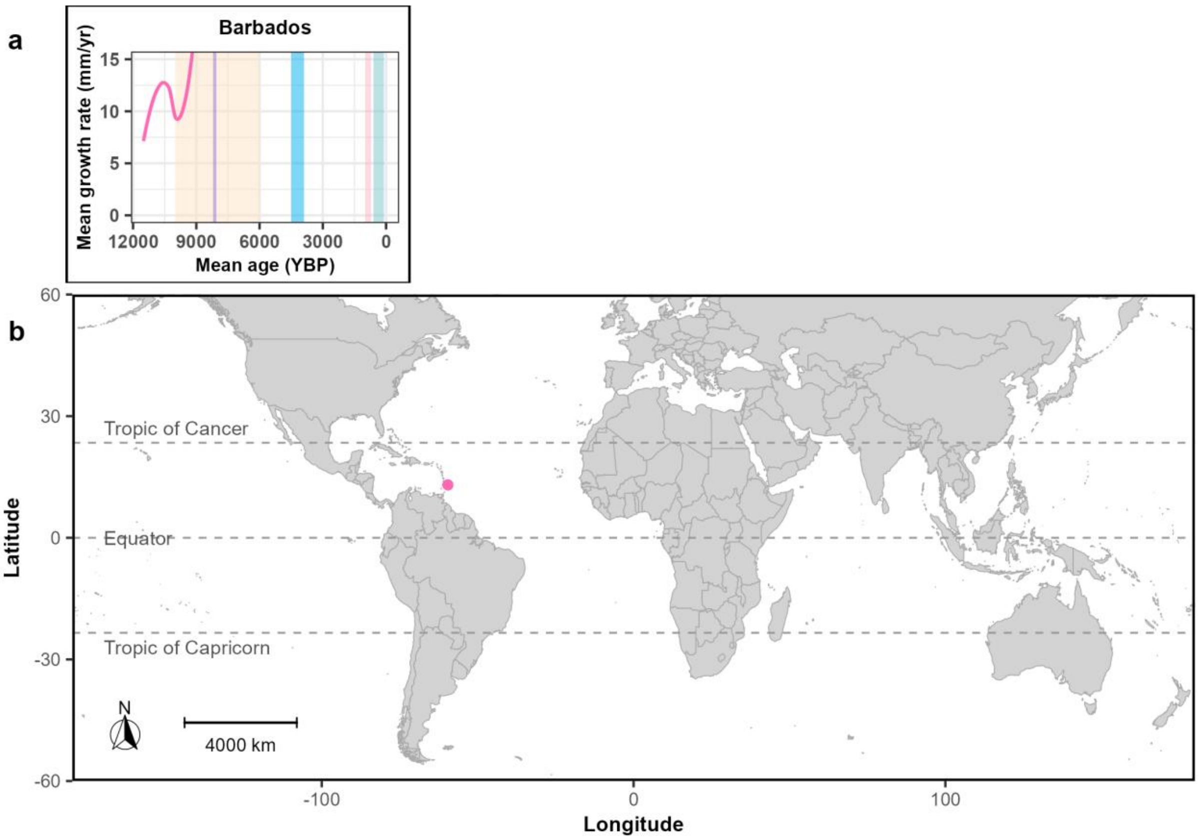

S13 Fig. (a) locally estimated scatterplot smoothing (LOESS) plot of mean coral-reef growth rate (mm/yr) data in the Barbados region during the Holocene, and (b) the site locations for the Barbados region on a world map as a pink point. The data were from 21 samples at 4 sites spanning from 11,700 years before present (BP; 1950 Common Era [CE]), through the Holocene. The basemap was created using world polygons from *maps* [19] in R.

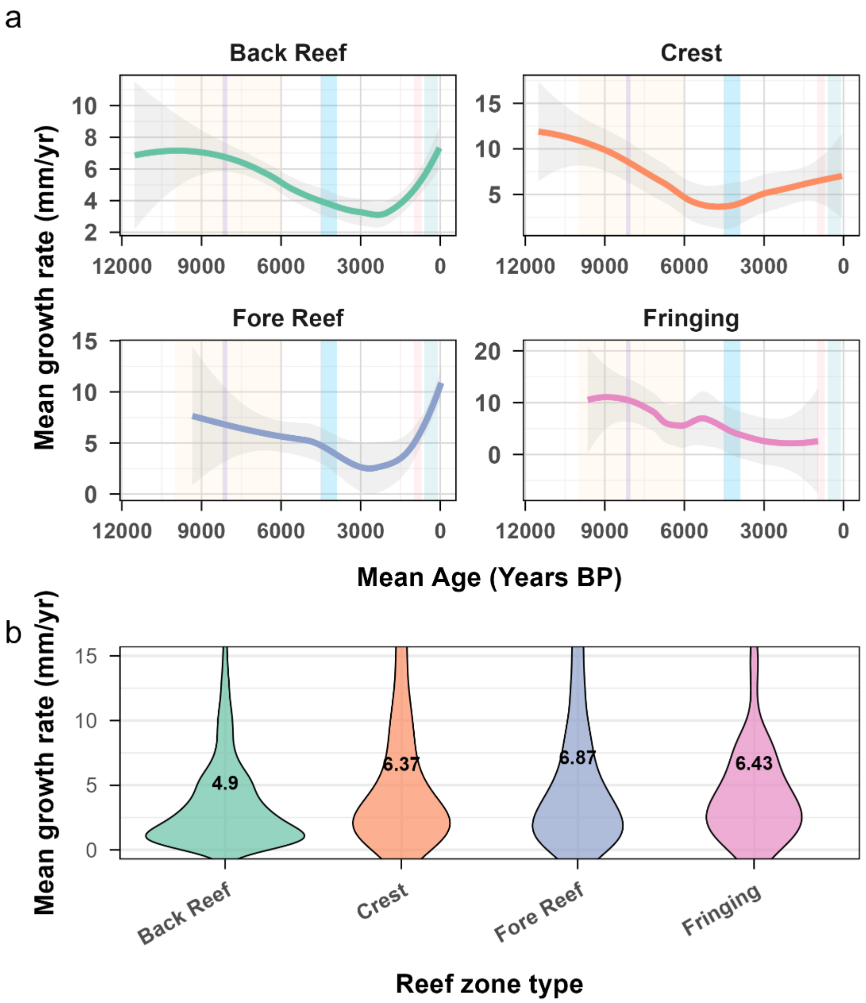

S14 Fig. (a) Mean coral-reef growth rates (mm/yr) across the Holocene years before present (BP; 1950 CE). The results are grouped by reef zone type and shown using locally estimated scatterplot smoothing (LOESS) for the back reef (green line), crest (orange line), fore reef (dark purple line), and fringing reef zones (dark pink line). Shaded areas indicate major climate events: Holocene Thermal Maximum (yellow), 8.2 ka yr BP cooling event (light purple), 4.2 ka yrs BP cooling event (blue), Medieval Climate Anomaly (light pink), and Little Ice Age (light blue). (b) Violin plots of mean coral-reef growth rate distributions (mm/yr) for each reef zone type, with the overall mean coral-reef growth rate displayed at its corresponding y-axis position within each violin. Reef zone classifications for back reef (green), crest (orange), fore reef (dark purple), and fringing reefs (dark pink) were obtained from the RADReef database [20]. Of 1,890 samples, 156 were listed as N/A, and 11 were classified as Mangrove, which were excluded, leaving 1,723 samples used to evaluate reef growth across the four reef zones at 291 sites across the Pacific, Indian, and Atlantic Oceans, from 11,700 years before present (BP; 1950 Common Era [CE]), through the Holocene.

Supporting Information References

1. Osman MB, Tierney JE, Zhu J, Tardif R, Hakim GJ, King J, et al. Globally resolved surface temperatures since the Last Glacial Maximum. Globally resolved surface temperatures since the Last Glacial Maximum. NOAA National Centers for Environmental Information; 2021. Available: (https://www.ncdc.noaa.gov/paleo/study/33112)

2. Toscano MA, Macintyre IG. Corrected western Atlantic sea-level curve for the last 11,000 years based on calibrated 14C dates from Acropora palmata framework and intertidal mangrove peat. Coral Reefs. 2003;22: 257–270.

3. Lambeck K. Shoreline reconstructions for the Persian Gulf since the last glacial maximum. Earth Planet Sci Lett. 1996;142: 43–57.

4. Wang F, Zong Y, Mauz B, Li J, Fang J, Tian L, et al. Holocene sea-level change on the central coast of Bohai Bay, China. Earth Surf Dyn. 2020;8: 679–693.

5. Fairbanks RG. A 17,000-year glacio-eustatic sea level record: influence of glacial melting rates on the Younger Dryas event and deep-ocean circulation. Nature. 1989;342: 637–642.

6. Lambeck K, Purcell A, Flemming NC, Vita-Finzi C, Alsharekh AM, Bailey GN. Sea level and shoreline reconstructions for the Red Sea: isostatic and tectonic considerations and implications for hominin migration out of Africa. Quat Sci Rev. 2011;30: 3542–3574.

7. Chua S, Switzer AD, Li T, Chen H, Christie M, Shaw TA, et al. A new Holocene sea-level record for Singapore. Holocene. 2021;31: 1376–1390.

8. Grossman EE, Fletcher CH. Sea level higher than present 3500 years ago on the northern main Hawaiian Islands. Geology. 1998;26: 363–366.

9. Loveson VJ, Nigam R. Reconstruction of late Pleistocene and Holocene sea level curve for the East Coast of India. J Geol Soc India. 2019;93: 507–514.

10. Nunn P. Coastal processes and landforms of Fiji: their bearing on Holocene sea-level changes in the South and West Pacific. J Coast Res. 1990;6: 279–310.

11. Nunn P. Sea levels, shorelines and settlements on Pacific reef islands. Archaeology in Oceania. 2016;51: 91–98.

12. Camoin GF, Colonna M, Montaggioni LF, Casanova J, Faure G, Thomassin BA. Holocene sea level changes and reef development in the southwestern Indian Ocean. Coral Reefs. 1997;16: 247–259.

13. Horton BP, Culver SJ, Hardbattle MIJ, Larcombe P, Milne GA, Morigi C, et al. Reconstructing Holocene sea-level change for the central Great Barrier Reef (Australia) using subtidal Foraminifera. J Foraminiferal Res. 2007;37: 327–343.

14. Khan NS, Ashe E, Shaw TA, Vacchi M, Walker J, Peltier WR, et al. Holocene relative sea-level changes from near-, intermediate-, and far-field locations. Curr Clim Change Rep. 2015;1: 247–262.

15. Wyrwoll K, Zhu Z, Kendrick G, Collins L, Eisenhauer A. Holocene sea-level events in Western Australia: revisiting old questions. Journal of Coastal Research. 1995; 321–326.

16. Jones LA, Gearty W, Allen BJ, Eichenseer K, Dean CD, Galván S, et al. palaeoverse: A community-driven R package to support palaeobiological analysis. Methods Ecol Evol. 2023;14: 2205–2215.

17. World Map Data from Natural Earth [R package rnaturalearth version 1.1.0]. In: Comprehensive R Archive Network (CRAN) [Internet]. 28 Jul 2025. Available: https://CRAN.R-project.org/package=rnaturalearth

18. R-INLA Project. Available: https://www.r-inla.org/

19. Becker RA, Wilks AR, Brownrigg R, Minka TP, Deckmyn A. Draw Geographical Maps [R package maps version 3.4.3]. In: Comprehensive R Archive Network (CRAN) [Internet]. 26 May 2025. Available: https://CRAN.R-project.org/package=map

20. Hynes MG, O'Dea A, Webster JM, Renema W. RADReef Dataset Files. RADReef: A global Holocene Reef Rate of Accretion Dataset. Figshare; 2024. doi:10.6084/m9.figshare.25251157.v2
